# Mapping regulatory variants controlling gene expression in drought response and tolerance in maize

**DOI:** 10.1186/s13059-020-02069-1

**Published:** 2020-07-06

**Authors:** Shengxue Liu, Cuiping Li, Hongwei Wang, Shuhui Wang, Shiping Yang, Xiaohu Liu, Jianbing Yan, Bailin Li, Mary Beatty, Gina Zastrow-Hayes, Shuhui Song, Feng Qin

**Affiliations:** 1 State Key Laboratory of Plant Physiology and Biochemistry, College of Biological Sciences, China Agricultural University, Beijing, 100193 China; 2 National Genomics Data Center & CAS Key Laboratory of Genome Sciences and Information, Beijing Institute of Genomics, Chinese Academy of Sciences, and China National Center for Bioinformation, Beijing, 100101 China; 3 Agricultural College, Yangtze University, Jingzhou, 434025 China; 4 National Key Laboratory of Crop Genetic Improvement, Huazhong Agricultural University, Wuhan, 430070 China; 5 Corteva Agriscience, Johnston, IA 50131 USA

**Keywords:** Regulatory variants, Gene expression, Drought response, Stress tolerance, Maize

## Abstract

**Background:**

Gene expression is a key determinant of cellular response. Natural variation in gene expression bridges genetic variation to phenotypic alteration. Identification of the regulatory variants controlling the gene expression in response to drought, a major environmental threat of crop production worldwide, is of great value for drought-tolerant gene identification.

**Results:**

A total of 627 RNA-seq analyses are performed for 224 maize accessions which represent a wide genetic diversity under three water regimes; 73,573 eQTLs are detected for about 30,000 expressing genes with high-density genome-wide single nucleotide polymorphisms, reflecting a comprehensive and dynamic genetic architecture of gene expression in response to drought. The regulatory variants controlling the gene expression constitutively or drought-dynamically are unraveled. Focusing on dynamic regulatory variants resolved to genes encoding transcription factors, a drought-responsive network reflecting a hierarchy of transcription factors and their target genes is built. Moreover, 97 genes are prioritized to associate with drought tolerance due to their expression variations through the Mendelian randomization analysis. One of the candidate genes, *Abscisic acid 8′-hydroxylase*, is verified to play a negative role in plant drought tolerance.

**Conclusions:**

This study unravels the effects of genetic variants on gene expression dynamics in drought response which allows us to better understand the role of distal and proximal genetic effects on gene expression and phenotypic plasticity. The prioritized drought-associated genes may serve as direct targets for functional investigation or allelic mining.

## Background

Maize is an important crop for food, forage, and industrial compounds [1, 2] but also serves as a model organism for its abundant genetic polymorphisms and quick linkage decay on a genome-wide scale [3]. Drought stress is a major environmental factor which limits crop production worldwide [4]; thus, understanding the molecular mechanisms and identifying the functional variants underlying drought tolerance are critical for the trait improvement. However, as a complex quantitative trait, genetic dissection for plant drought tolerance is difficult. Although genome-wide association studies (GWAS) facilitate the detection of genetic and phenotypic associations, identification of the causal genes or DNA variants that mechanistically affect the trait is still challenging [5, 6]. Cumulative evidence suggests that genetic variants influence complex traits through modulating gene expression; highlighting the functional importance in regulatory variants. For instance, a short interspersed nuclear element (SINE), *AluJb*, located 20 kb upstream of *lin-28 homolog B* (*LIN28B*) drives the majority of *LIN28B* expression in a substantial number of human tumors for oncogene activation [7]. *Teosinte branched 1* (*Tb1*) gene expression is activated due to a transposon insertion 60 kb upstream of *Tb1*, which contributes to maize domestication from its wild ancestor teosinte (*Zea mays* ssp. *parviglumis*) [8]. A genetic insertion upstream of in *Vegetative to generative transition 1* (*vgt1*) represses gene expression and promotes maize flowering time [9]. Genetic variations in *ZmVPP1* and *ZmNAC111I* gene regulatory regions affect gene expression which are associated with the natural variation in drought tolerance [6, 10]. Recently, a 2-bp deletion, 9.5 kb upstream of *ZmRAVL1*, was identified as the causal variant responsible for the enlarged leaf angles in maize [11].

The genetic basis of gene expression variation, also known as quantitative trait loci (eQTLs), was studied in many model organisms [12–14]. However, a classical strategy to identify the genetic determinants for gene expression through analyzing a population of recombinant offspring of two parent strains usually detects large genomic intervals lacking accurate information for causal gene prediction. Recently, association analysis, based on genetic linkage disequilibrium (LD) at a natural population level, exploits historical and evolutionary recombination with high-density markers, providing an alternative strategy for eQTL dissection [15, 16]. Due to the quick linkage disequilibrium (LD) decay in the maize genome, resolution of the detected regions in association studies often reaches a single-gene level [6, 17]. Moreover, given that gene expression is spatio-temporal dependent, eQTL architecture can change dynamically during development or in response to environmental stimuli. Such dynamics are considered to play an important functional role and have been thoroughly explored in multiple types of human tissues [18], but rarely in other species.

More importantly, gene expression alterations are regarded as important molecular phenotypes that link genotypes to molecular functions and plant fitness, whose regulation is of fundamental importance to stress response and adaptation [19]. Strategies, such as Mendelian randomization (MR) analysis and transcriptome-wide association study (TWAS), were recently developed to identify expression-trait associations, which prioritize candidate genes associated with the trait due to gene expression variations [20, 21]. Thus, a comprehensive and accurate understanding of the plasticity of gene expression architecture in response to stress is essential but demanding.

In this research, 627 RNA-seq data of 224 maize accessions (inbred lines) subjected to three water regimes were generated. A genome-wide association study (GWAS) was performed for ~ 30,000 expressed genes under each condition. In total, 73,573 eQTLs were detected, and 60% of them were resolved to a single candidate gene. Importantly, MR analysis prioritized 97 genes that contribute to drought tolerance due to gene expression variations.

## Results

### RNA-seq analysis of 224 maize accessions subjected to three water regimes

**Fig. 1:**
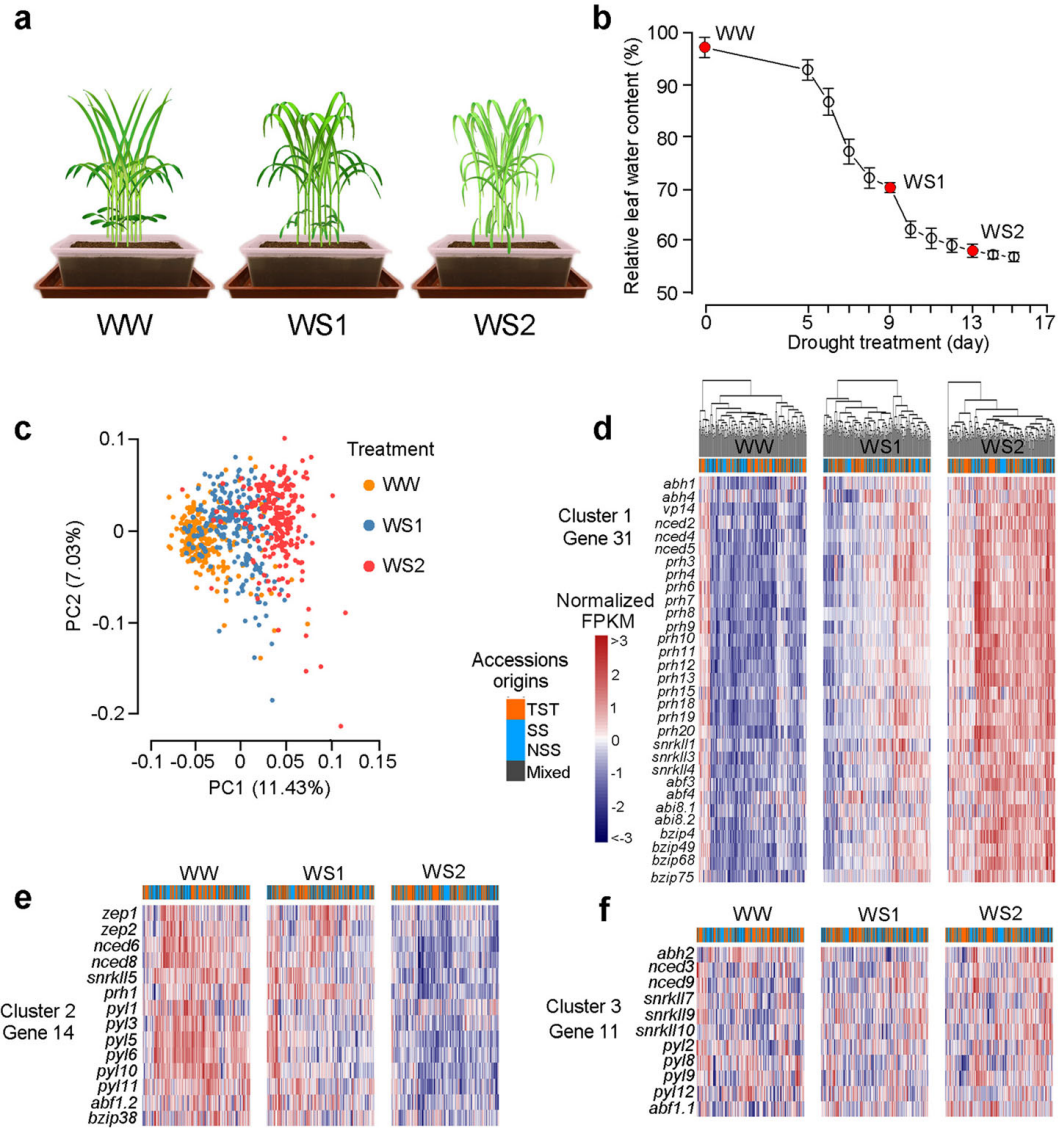
Drought treatment, transcriptome analysis, and ABA-related gene expression profiling across 224 maize accessions. **a** Illustration of the drought treatment and typical appearance of seedlings subjected to WW (well-watered), WS1 (moderate drought), and WS2 (severe drought) water regimes. **b** The representative curve of relative leaf water content (RLWC) during the drought treatment and at the points at which WW, WS1, and WS2 plants were sampled. Error bars and s.d. (standard deviation) were from the above three biological replicates. **c** Principal component analysis (PCA) of the 627 transcriptomes. **d**–**f** Clustering analysis of the regulation pattern of ABA-related genes among 224 maize accessions. The color bar indicates the normalized gene expression. The gene expression patterns were sorted into three clusters by *k*-means clustering algorithm. Cluster 1: drought-inducible genes (**d**). Cluster 2: drought-repressed genes (**e**). Cluster 3: response variable genes (**f**). The transcriptomic hierarchies of all the accessions were constructed based on the gene expression levels of 56 ABA-related genes. They were presented on the top of cluster 1. The origin of maize accessions is indicated by the bars across the top of each panel: orange for TST (tropical/subtropical); blue for stiff stock and non-stiff stock (SS and NSS); gray for mixed origins

To understand how genetic variants affect drought-responsive gene expression on a whole transcriptome scale in maize seedlings, a collection of 224 accessions was assembled from a previously characterized population that had been used for the genetic dissection of a number of agronomic traits [17, 22–24]. These accessions were selected with the maximum retention of phenotypic diversity in regard to seedling viability under drought to achieve a wide coverage of phenotypic diversity [6] (Additional file 1: Figure S1a). At the meantime, this collection retained the genetic diversity of the original population which consists of the accessions with the tropical/subtropical (TST), temperate (including stiff stalk and non-stiff stalk), and mixed origins (Additional file 1: Figure S1b). Watering was withheld from soil-growing seedlings at the Vegetative 2–3 stage, and the decline in relative leaf water content (RLWC) was recorded. Leaf samples were collected under well-watered (WW) and the conditions when RLWC dropped to ~ 70% (WS1) and ~ 58% (WS2), respectively (Fig. 1a, b). The treatment and typical plant appearance under well-watered (WW), WS1, and WS2 treatments were illustrated in Fig. 1a. All samples were subjected to RNA-seq analyses. In total, 32.48 billion high-quality reads were generated, averaging 44.1 million reads per sample (Additional file 1: Figure S1c). Samples with less than 10 million reads were omitted from downstream analyses to achieve a reliable quantification of gene expression based on the unique mapped reads to the B73 reference genome RefGen_V4 [25]. As a result, 627 transcriptomes were obtained for 224 maize accessions subjected to the WW (209), WS1 (208), and WS2 (210) treatments. The single nucleotide polymorphisms (SNPs) from the yielded reads were also called for genotype confirmation and to complement previously published data on genotypic variation [16]. The average concordant rate of the genotyping data of this study and the previous work was 93.7% (Additional file 2: Table S1). To avoid any uncertainty, we designated the sites as “data missing” where inconsistent genotype data were observed. In total, 1,288,889 SNPs were obtained for the 224 maize accessions, with a missing rate < 0.6 and a minor allele frequency ≥ 5% (Additional file 1: Figure S1d). The SNPs were later used for eQTL identification.

### Abscisic acid-related genes were differentially expressed in response to drought among the distinct genotypes

Principal component analysis (PCA) was performed on the 627 transcriptomes to analyze transcriptome-wide response to different levels of water stress. Each transcriptome was represented by the expression profiles of 15,369 DEGs (genes that were differentially expressed between any of the two treatments and detected in greater than 20% of the accessions). Generally, transcriptomic changes were gradually observed with the increase in water stress. This indicates that the water stress treatments effectively induced changes in the gene expression (Fig. 1c). Using the *k*-means clustering algorithm, we classified the 15,369 DEGs into 3 clusters (Additional file 1: Figure S2a). In addition to the up- and downregulated gene clusters, 4254 genes (27.7%) exhibited variable drought-responsive patterns among these accessions, indicating the response diversities in different genetic backgrounds. This cluster of genes was especially enriched in the Gene Ontology terms of response to various stresses or hormone stimuli, inferring that natural variants played a clear regulatory role on these gene expressions among the different maize accessions (Additional file 1: Figure S2b).

Since the phytohormone abscisic acid (ABA) plays a vital role in environmental stress response, we were particularly interested in gene expression variations related to ABA signaling and biosynthesis in response to the stress in the different accessions. Clustering of gene expression pattern indicated that among 56 ABA-related DEGs, 31 were generally upregulated in all the different accessions, including genes for ABA biosynthesis (*vp14*, *nced2*, *nced4*, and *nced5*), ABA catabolism (*abh1* and *abh4*), ABA co-receptors (fourteen *protein phosphate 2C* genes, *prhs*), protein kinase in ABA signaling (*snrkII1*, *snrkII3*, *snrkII4*), and transcription factors (*bzip4*, *bzip49*, *bzip68*, *bizp75*, *abf3*, *abf4*, *abi8.1*, and *abi8.2*) (Fig. 1d). Fourteen genes were generally downregulated by water stress, including genes encoding ABA receptors (*pyl1*, *pyl3*, *pyl5*, *pyl6*, *pyl10*, and *pyl11*); *zep1* and *zep2*, encoding the first enzyme in ABA biosynthesis (zeaxanthin epoxidase); and *nced6* and *nced8*, encoding a rate-limiting enzyme for ABA biosynthesis (Fig. 1e). It possibly reflected a feedback of response to avoid over-reaction to the signal, which has also been observed in other phytohormone responses, such as gibberellin and brassinosteroid [26, 27]. Interestingly, eleven genes, *abh2*, *nced3*, *nced9*, *snrkII7*, *snrkII9*, *snrkII10*, *pyl2*, *pyl8*, *pyl9*, *pyl12*, and *abf1.1*, exhibited diversity in response direction among the investigated accessions (Fig. 1f). The diversity in response intensity and direction indicated that natural variants underlie the expression of these genes and potentially contribute to the variation of drought response among these accessions. In addition, accessions having the same origin apparently did not cluster together based on their gene expression patterns, indicating that variations in gene expression were not completely dependent on the similarity of their genomes and were also environmentally dependent (Fig. 1d–f, Additional file 1: Figure S2a).

### Static and dynamic eQTLs were identified in response to drought

**Fig. 2:**
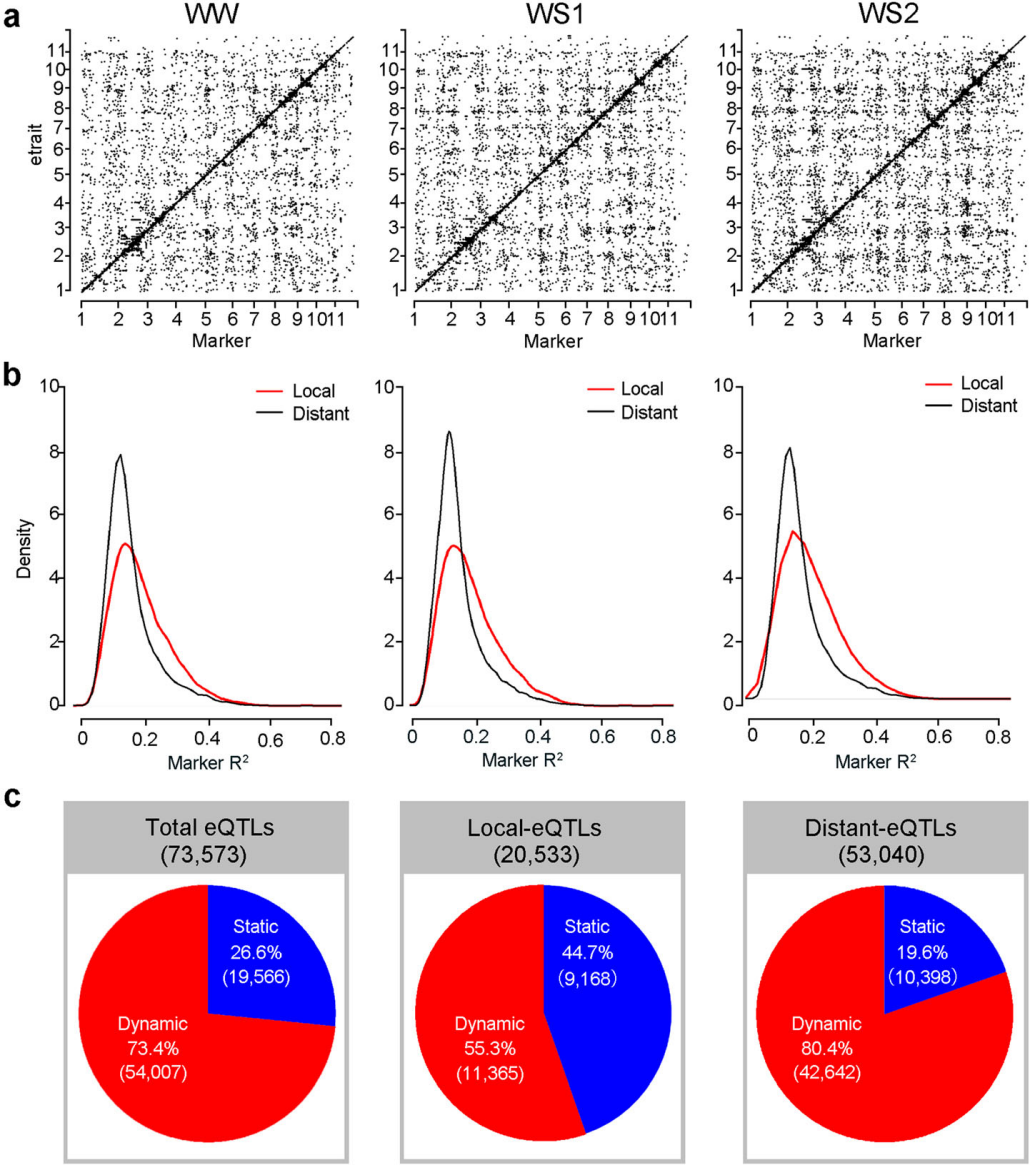
Large-scale local and distant eQTLs identified by GWAS. **a** Genome distribution of eQTLs (*x*-axis) and the etrait genes (*y*-axis). The start position of the mapped genes in the maize genome is plotted against the position of the lead SNPs for the eQTLs. Chromosome 11 was the B73_V4_contig. **b** The distribution of the effects of local and distant eQTLs. **c** From left to right, percentage of static and dynamic eQTLs in the total, local, and distant eQTLs

Expression GWAS was conducted to identify genetic variants controlling gene expressions, in which the variation in gene expression in the population was treated as a quantitative trait (etrait) and its association with the 1.28M genetic variants was tested. To avoid random errors, the determination of an eQTL was only recognized when at least three significant SNPs were detected in association with each treatment (Benjamini-Hochberg rejection, FDR < 0.05). The identified eQTL was categorized based on the most significant SNP. Resultantly, when the start positions of the etrait gene were plotted against the position of the lead SNP of its detected eQTL, a plotted line along the diagonal was observed reflecting the local regulations (Fig. 2a). The eQTLs with lead SNPs located within 20 kb up- or downstream of the etrait gene were defined as local eQTLs, while all others were regarded as distant eQTLs. A comparison of the determination coefficients of the two types of eQTLs demonstrated that local eQTLs tend to have larger effects than distant eQTLs in all three water regime treatments, indicating a greater local effect on gene expression regulation (Fig. 2b). A total of 73,573 eQTLs (including 23,771 in WW, 22,945 in WS1, and 26,857 in WS2) were detected capturing an unprecedented range of regulatory variants controlling gene expression in response to drought stress (Additional file 2: Table S2). According to eQTL occurrence in different treatments, the eQTLs for an etrait that were consistently detected in all three treatments were regarded as static eQTLs, while eQTLs detected in only one or two treatments were designated as dynamic eQTLs in response to drought. Thus, 19,566 (26.6%) were identified as static and 54,007 (73.4%) as dynamic eQTLs (Fig. 2c).

**Fig. 3:**
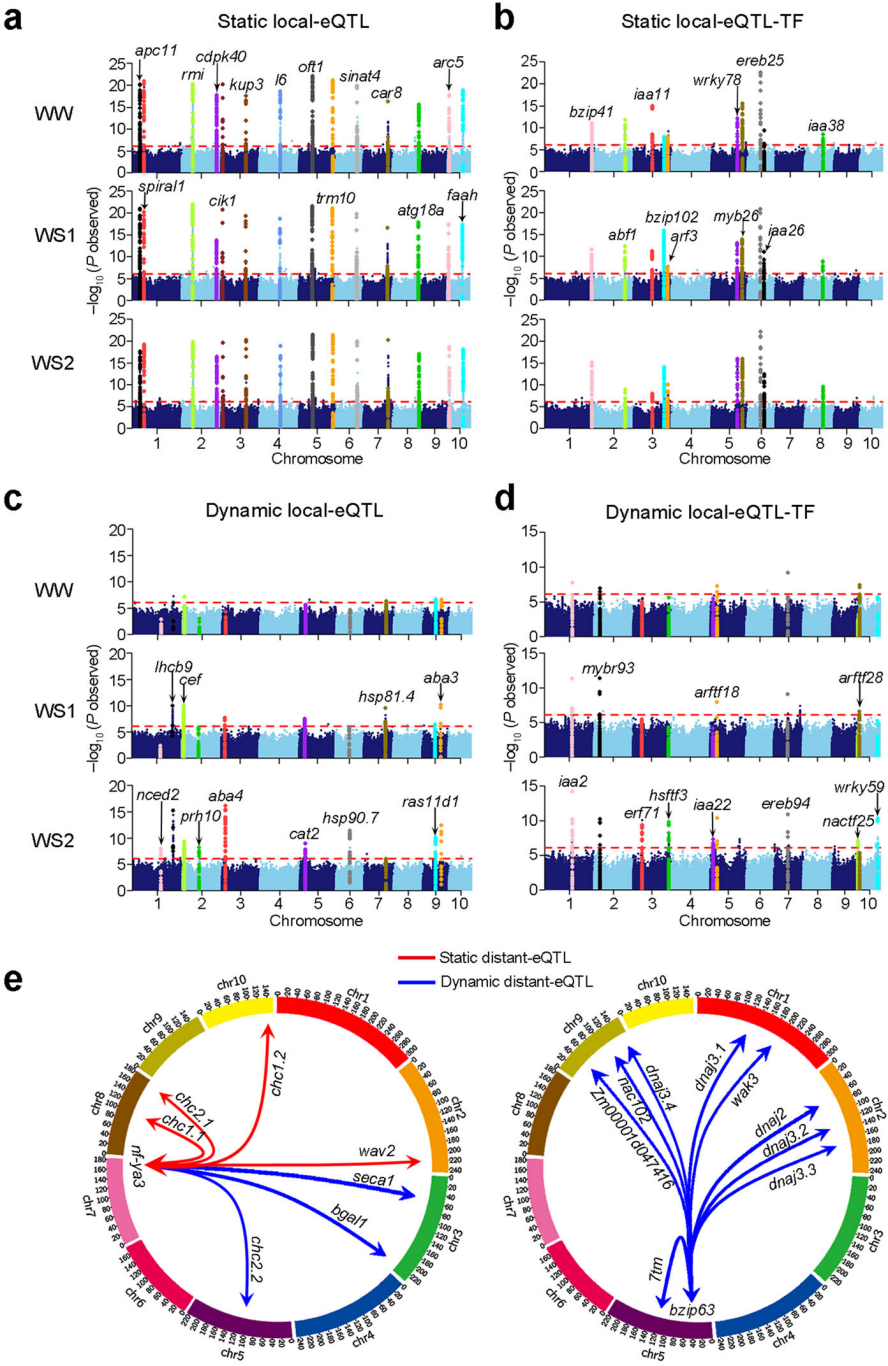
Examples of static and dynamic eQTLs reflect how the detected local and distant regulatory variants affect the etrait gene expression under WW or WS conditions. **a** Combined Manhattan plot for different static, local eQTLs detected for individual etraits; demonstrating local genetic variants are significantly associated with the etrait gene expression under the three different water regimes. **b** Combined Manhattan plot for static local eQTLs of which the etrait genes encode *TF* genes. **c** Combined Manhattan plot for dynamic local eQTLs demonstrating a significant association of local genetic variants with the etrait gene expression only detectable under drought stress. **d** Manhattan plot for dynamic local eQTLs of which the etrait genes encode *TF* genes. Different colors represent an individual etrait. **e** Left panel: natural variation in *nf-ya3* function as distant eQTLs underlying several target gene expressions and also local eQTL for its own expression under all conditions. Right panel: natural variation in *bzip63* function as dynamic distant eQTLs underlying nine target gene expressions and also local eQTL for its own expression under WW and/or WS

As for local regulatory variants, 9168 static local eQTLs (44.7% of total 20,533 local eQTLs) were repeatedly and stably identified for 3056 genes, based on the gene expression data independently obtained from the three treatments (Fig. 2c, Additional file 2: Table S2). For example, very significant and unique association peaks were repeatedly identified in the three water regimes respectively for genes involved in plant growth and development (*apc11*, *spiral1*, *oft1*, *cik1*, *rmi*, *arf3*, *iaa11*, *iaa26*, and *iaa38*), signal transduction (*cdpk40*, *car8*, and *sinat4*), potassium transport (*kup3*), ribosomal protein (*l6*), tRNA nucleoside modifier (*trm10*), and transcription regulation (*bzip41*, *bzip102*, *myb26*, and *abf1*) (Fig. 3a, b). The results provide strong evidence that these local regulatory variations significantly affect their own or adjacent (within 20 kb) gene expressions. Notably, among 11,365 dynamic local eQTLs, 5492 were only detected under the drought stress treatments Additional file 2: Table S2. They include genes involved in oxidative detoxification (*cat2* and *nactf25*), photosynthesis (*lhcb9*), protein trafficking (*ras11d1*), calcium binding (*cef*), ABA response (*nced2*, *prh10*, *aba3*, and *aba4*), stress response (*hsp81.4*, *hsp90.7*, *hsftf3*, *erf71*, *ereb94*), and transcription regulation (*iaa2*, *iaa22*, *arftf18*, *arftf28*, *wrky59*, *mybr93*). The association signals were not detectable under well-watered conditions but remarkably increased upon the stress treatment, and some of them were enhanced in accordance with the increase of the stress (Fig. 3c, d). These data infer that local variants potentially confer the stress responsiveness on these genes among the different maize accessions.

As for distant regulatory variants, 10,398 static distant eQTLs and 42,642 dynamic distant eQTLs were identified, revealing a massive and dynamic regulatory network in maize seedlings (Fig. 2c, Additional file 2: Table S2). Only 19.6% distant eQTLs were static, compared with 44.7% local eQTLs, indicating that distant genetic variants exert a more dynamic regulatory role than local eQTLs on gene expression. Several eQTL hotspots were resolved to genes encoding transcription factors (TFs). For example, a number of gene expressions were associated with natural variants in *nf-ya3*, encoding a nuclear factor-Y TF, which plays an important role in the regulation of photoperiod-dependent flowering and drought stress in maize [28]. *chc1.1* (*Clathrin Heavy Chain1.1*), *chc1.2*, *chc2.1*, and *wav2* (*wavy growth 2*), which play important roles in stomatal movement, gas exchange, and root bending in *Arabidopsis* [29], were identified as *nf-ya3* target genes in all three water regimes (Fig. 3e). *seca1* (*chloroplast Sec translocation machinery ATPase subunit 1*), which is essential for chloroplast biogenesis and the regulation of photosynthetic complexes in *Arabidopsis* [30], and *bgal1* (*β-galactosidases 1*), which contributes to the maintenance of cell wall architecture during cell elongation [31], were dynamically regulated by *nf-ya3* (Fig. 3e). Another example of a distant eQTL that functions as an eQTL hotspot is the *bZIP TF*, *bzip63*, which has been reported as an important node in the glucose-ABA interaction network [32]. *DnaJ homologs* (*dnaj2*, *dnaj3.1*, *dnaj3.2*, *dnaj3.3*, *dnaj3.4*), *7tm* (*seven-transmembrane helix*), *nac102*, *wak3* (*Wall-associated kinase 3*), and *Zm00001d047416* were dynamically identified as target genes of *bzip63*. *dnaj3* encodes a chaperone which has been reported to play a positive role in plant salinity and alkalinity tolerance in *Arabidopsis* [33].

### Regulatory hierarchy based on dynamic distant eQTLs resolved to *TF* genes and drought-related target genes

**Fig. 4:**
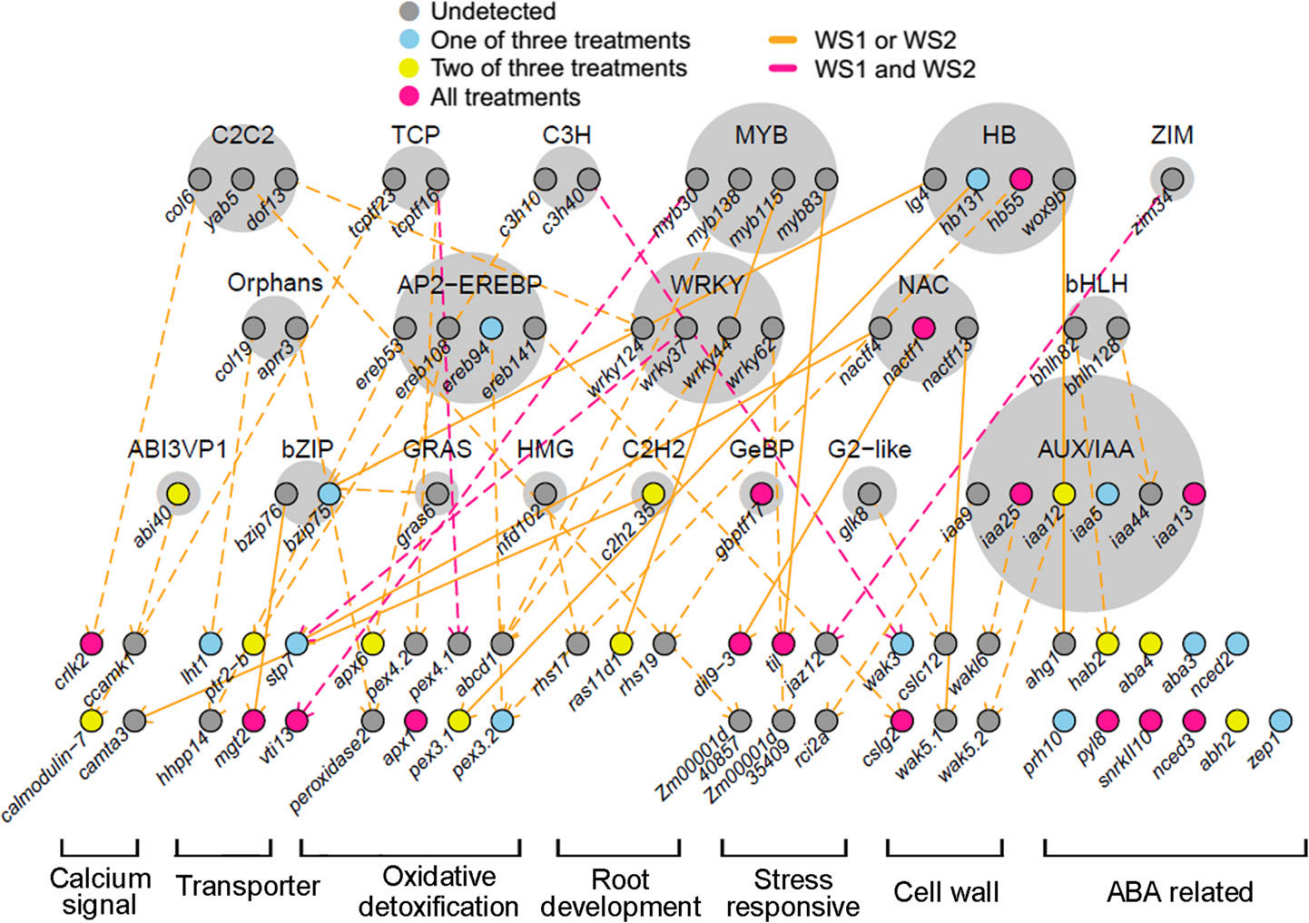
A network model of TF hierarchy decoded by dynamic distant eQTLs and their regulated genes. TFs are arranged in three tiers according to normalized hierarchy height. Target genes are grouped by biological function. Dashed lines indicate dynamic distant eQTL and its target genes. Colored circles indicate local eQTLs that were detected for these genes. Solid lines indicate that the regulatory relations are supported by a published ChIP-seq analysis [35]

One of the most plausible modes of action for distant eQTLs is the involvement of transcription factors that regulate target gene expression in a *trans*-acting fashion. Therefore, we were interested in the distant eQTLs resolved to TF-encoding genes. Among all 73,573 eQTLs, the lead SNPs of 3733 eQTLs directly located in *TF* genes which belong to 66 TF gene families (Additional file 2: Table S3). Gene enrichment analysis revealed that bZIP, NAC, AP2/EREBP, WRKY, and MYB were the top five TF families, indicating that their genetic variants play a major role in regulating the gene expression in maize (Additional file 1: Figure S3a). Due to our interest in the regulation of drought-responsive gene expression, we focused on the dynamic eQTLs resolved to *TF* genes and their etraits (target genes). Based on the previous knowledge on plant drought response and adaptation [34], we focused on the regulation of 44 etraits involved in different aspects of cellular response, including calcium signaling, transport, oxidative detoxification, root development, stress response, cell wall formation, and ABA signaling and biosynthesis. A drought-responsive regulatory network was constructed based on the relationship of the dynamic eQTLs (encoding TFs) and these etraits, providing a model of drought adaptation network regulated by local and distant genetic variations. A total of 45 individual *TF* genes, belonging to 19 TF families, were associated with 41 dynamic distant regulations of these etraits. In addition, local eQTLs were also identified for 12 *TF* genes and 26 etraits (Fig. 4, (Additional file 2: Table S4). Notably, ten of the distant regulatory activities, imparted by *MYB*, *NAC*, *bZIP*, *C2H2*, and *HB* TF family genes, were previously identified by chromatin immunoprecipitation sequencing (ChIP-seq) analysis using transgenic *Arabidopsis* [35], providing strong evidence for our distant eQTL identification (Additional file 2: Table S4). Since the expression of *TF* genes can also be regulated by distant eQTLs resolved to other *TFs*, a regulatory hierarchy of these TF gene families can be constructed based on their regulatory relationships. As a result, 19 TF gene families were organized into three tiers according to their hierarchical height. For example, the hierarchy position of AP2-EREBP, WRKY, and NAC families was lower than the HB and MYB families based on the ratio of the gene number of *TF* gene family members regulated to the number of *TFs* that regulate them. A similar regulatory hierarchy was also detected in studies of *Arabidopsis* and *Populus* [35, 36]. Regulatory relationships were also identified for some less-reported, drought-related TFs, such as TCP, C3H, and AUX/IAA, which provides valuable information for further studies. For example, two tandem *Peroxin* genes, *pex4.1* and *pex4.2*, are potentially regulated by tcptf16, a TCP family TF. The expression of two *Wall-associated kinase*, *wak5.2* and *wakl6*, may be controlled by two IAA TFs (Fig. 4).

### Mendelian randomization test prioritized 97 genes associated with drought tolerance

**Fig. 5:**
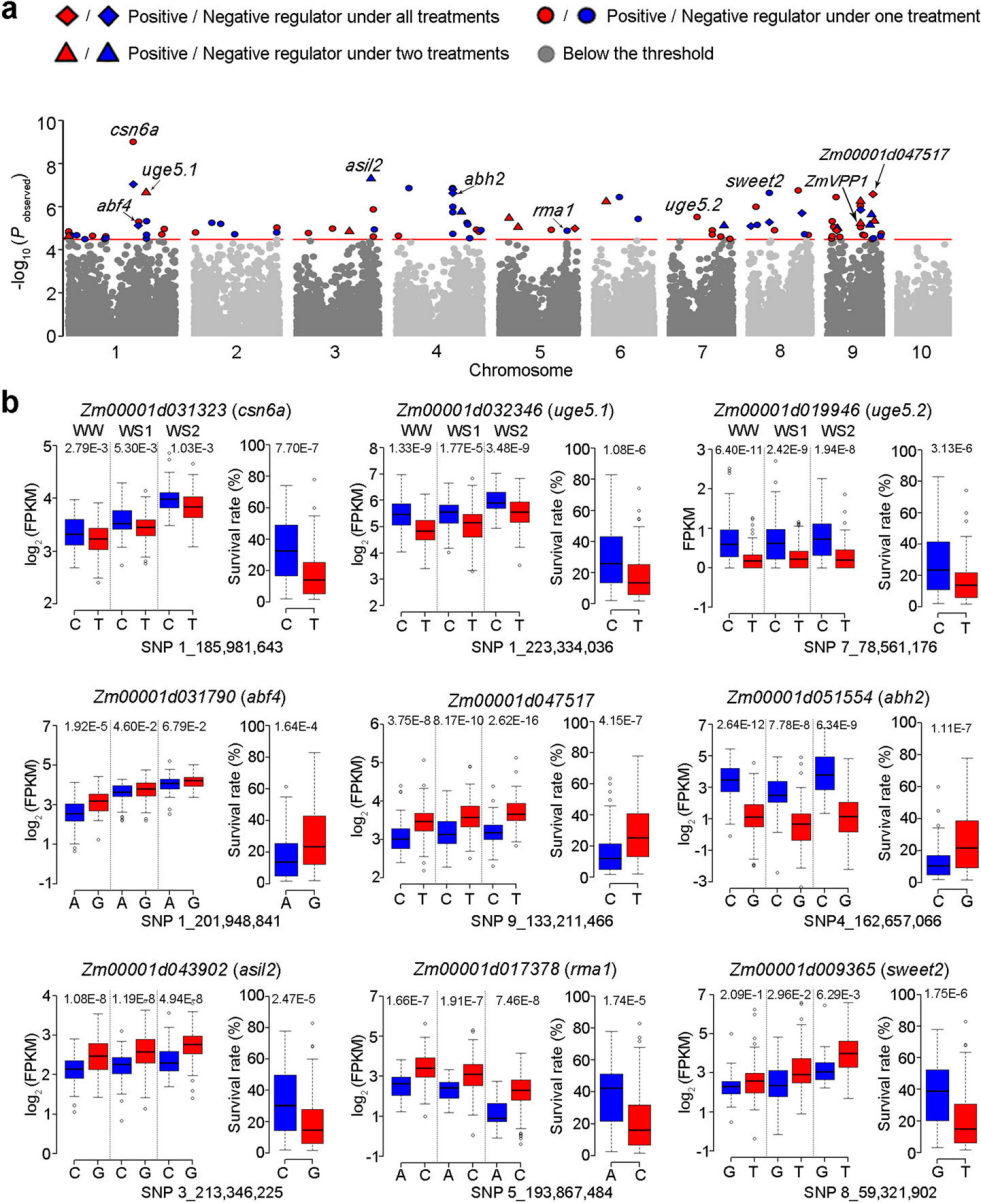
MR tests for the association between the gene expression and a drought tolerance phenotype. **a** Manhattan plot for the MR test. Each point represents an individual gene. The *y*-axis is the log_10_ (*P*) value of MR test significance. The most significant SNP for each gene among the three water regimes is presented. **b** Allelic comparison of gene expression level and survival rate for nine candidate genes (indicated by arrows in **a**) based on the lead SNP of eQTLs

Based on the newly obtained data on gene expression and the previous phenotypic measurements of drought tolerance of these accessions [6], we performed Mendelian randomization (MR) test [21] to identify genes whose expression levels (with identified regulatory variants) are relevant to plant drought tolerance. As a result, 97 candidate genes were prioritized for their contribution to drought tolerance based on their alterations in gene expression (Bonferroni threshold, *P* < 3.33 × 10^−5^). The expression of 51 genes was found to positively contribute to drought tolerance, and 46 were negatively associated with the trait (Fig. 5a, Additional file 2: Table S5). The positive regulators were identified for plant viability because their expression variations were positively related to plant survival rate under drought (Fig. 5b). Among them, *ZmVPP1* gene expression was found to be regulated by a dynamic local eQTL, and the expression was positively related with drought tolerance, which was consistent with the result from previous GWAS for drought-tolerant gene in maize seedling [6], supporting the present analytic strategy and statistical power of the MR analysis (Fig. 5a, Additional file 2: Table S5). Other identified positive regulators for drought tolerance (Fig. 5b) include the following: *Zm00001d031323* (*csn6a*) is a homologoue of *Arabidopsis CSN6A* (COP9 signalosome subunit 6A) which plays pleiotropic roles in plant development by regulating ubiquitination-mediated protein degradation [37]; *Zm00001d031790* (*abf4*), a homolog of *Arabidopsis ABF4*, which plays an important role in drought stress- and ABA-responsive gene expression [38]; *Zm00001d032346* and *Zm00001d019946* (*uge5.1* and *uge5.2*) are paralogous genes encoding UDP-D-glucose 4-epimerase (UGE) which interconverts UDP-glucose and UDP-galactose. UGEs function in cell wall carbohydrate biosynthesis and possibly in stress tolerance by supplying UDP-galactose for galactinol biosynthesis which was considered to act as an osmoprotectant in abiotic stress tolerance in *Arabidopsis* [39]; and *Zm00001d047517* encoding a reticulon-like protein, and its homolog in mammals was reported functioning in cell apoptosis and development [40].

As for the negative regulators, the regulatory variants and gene expression levels of the following genes were exemplified that they were negatively associated with plant drought tolerance (Fig. 5b, Additional file 2: Table S5). *Zm00001d051554* (*abh2*) encodes an abscisic acid 8′-hydroxylase (ABAox), which catalyzes the first step in the oxidative degradation of ABA, and in *Arabidopsis*, it promotes stomatal opening in response to high humidity [41]. *Zm00001d043902* (*asil2*) encodes a trihelix-transcription factor, and the *Arabidopsis* homolog represses seed maturation and desiccation-tolerant gene expression in vegetative tissues [42]. *Zm00001d017378* (*rma1*) encodes an E3 ubiquitin-protein ligase, similar to *Arabidopsis* RMA1, which plays a critical role in the proteolysis of plasma membrane aquaporin in response to dehydration [43]. *Zm00001d009365* (*sweet2*) encodes a putative sugar transporter, and its homologs in *Arabidopsis* and rice are characterized as hexose transporters on tonoplasts that are responsible for the transport of excess glucose from the cytosol into vacuoles [44]. All the prioritized genes represent valuable candidates for future functional validation and allelic mining.

### Local variants regulate *abh2* expression and negatively associated with drought tolerance

**Fig. 6:**
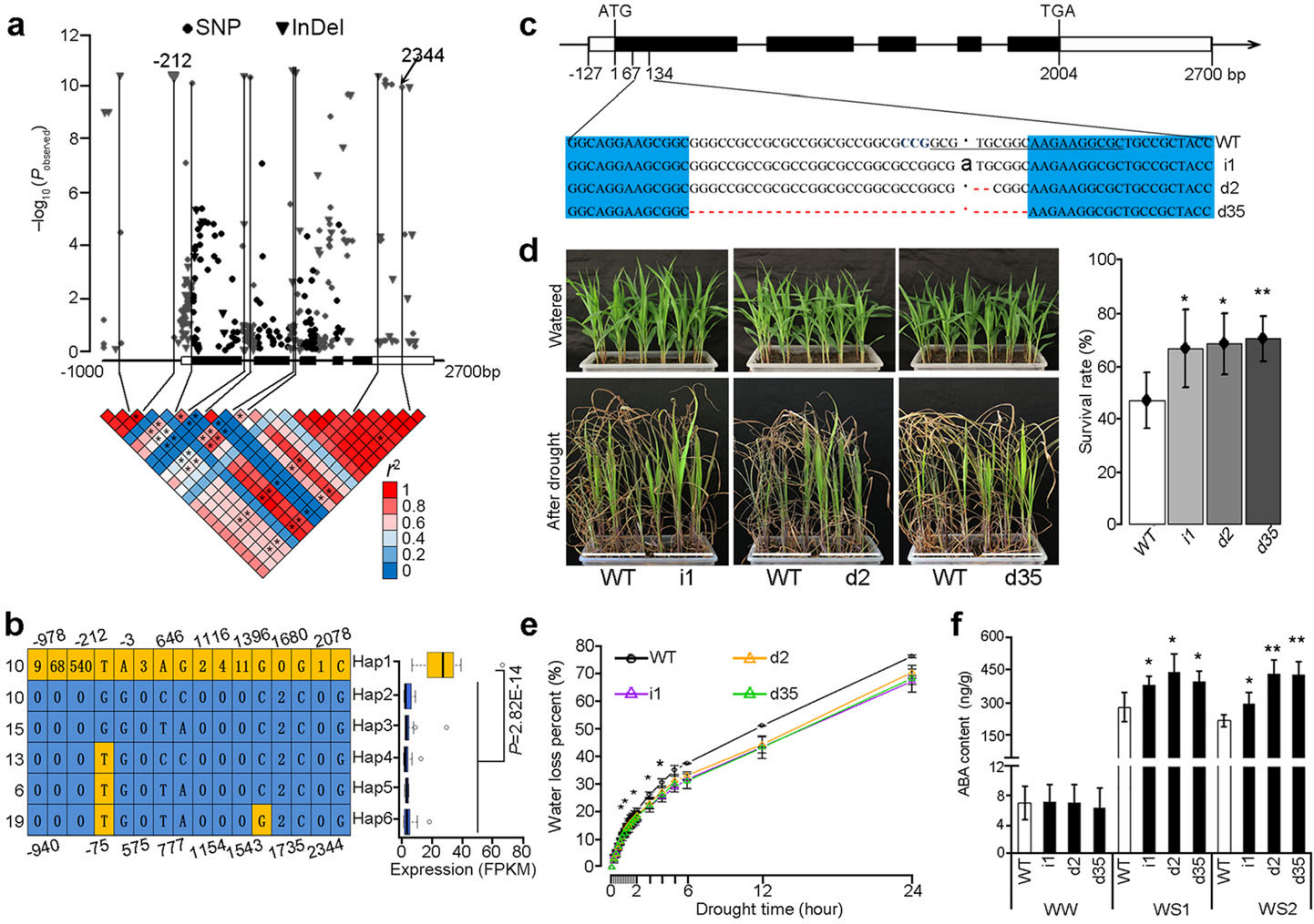
Validation of *abh2* local variants affecting the gene expression and the negative association of *abh2* with drought tolerance. **a***abh2*-based association mapping and pairwise linkage disequilibrium (LD) analysis for the variants of *P* < 10^−6^. The lead SNP of the *abh2* local eQTL (SNP 2334) in the 3′-UTR and newly identified variants (*P* < 10^−10^) are connected to the pairwise LD diagram with solid lines. Asterisks indicate the LD between these variants. **b** Six haplotypes of *abh2* were defined by the variants (*P* < 10^−6^). Gene expression levels of each haplotype group are displayed in the box plot, and the accession number of each haplotype was labeled in the left. Yellow color indicates the genotype of Haplotype1, and the blue color indicates the counterparts. **c** DNA sequence alignment of *abh2*-CRISPR locus of three homozygous lines. **d** Drought test of three *abh2*-CRISPR lines. Photographs were taken under well-watered conditions and after the stress treatment. Eighteen plants were tested for each genotype in each experiment. Statistical data were obtained from four replicates. **e** Rate of leaf water loss. Six leaves of each genotype were measured in each experiment, with three replicates. **f** ABA content quantification, with three replicates. Error bars, s.d.; statistical significance was determined by a two-sided *t* test: *P < 0.05, **P < 0.01

To verify the abovementioned findings, a 3.5-kb genomic region of *abh2* was sequenced among 141 maize inbred lines to check whether *abh2* expression levels were affected by local regulatory variants, and its expression was negatively related with drought tolerance (Fig. 5a, Additional file 2: Table S5). In total, 265 SNPs and 76 insertions or deletions (InDels) were identified, and all the significant variants (*P* < 1 × 10^−6^) were synonymous or locating in non-coding regions (Fig. 6a). Six major haplotype groups were identified based on significant variants (*P* < 1 × 10^−6^, accession number > 5, Fig. 6b). Haplotype 1 containing SNP2344 (C-genotype), the lead SNP identified as the *abh2* local eQTL, had significantly higher *abh2* gene expression than other haplotypes, supporting that local genetic variants affect the *abh2* expression (Fig. 5b). Since *abh2* expression was identified to be negatively associated with plant drought tolerance, the Clustered Regularly Interspaced Short Palindromic Repeats (CRISPR)-Cas9 technology was employed to generate the *ahb2*-CRISPR knockout plants. Three independent homozygous lines (i1, d2, and d35) were obtained (Fig. 6c), and their drought tolerance was compared with wild-type plants. The viability of all three mutants was significantly higher than wild-type plants after drought, supporting a negative role of *abh2* in stress tolerance (Fig. 6d). Less water loss was observed in detached leaves of the *abh2*-CRISPR lines over the course of a 1- to 4-h dehydration treatment, indicating that the *abh2* knockout potentially conferred a quicker stomatal closure in response to dehydration stress (Fig. 6e). When ABA content was quantified, the three CRIPSR lines exhibited approximately a 30% increase in ABA content compared to the wild type in the WS1 and WS2 treatments, suggesting that interfering *abh2* function may sustain a higher level of ABA when plants were challenged by stress (Fig. 6f). Collectively, the results support the implications of the eQTL and MR analyses. The identified natural variation in *abh2* provides a favorable allele for plant drought tolerance by modulating ABA content in response to water deficit.

## Discussion

In the present study, 73,573 eQTLs (regulatory variants) were identified that are responsible for the variation in the expression of ~ 30,000 genes during drought response. The degree of LD is a major factor affecting the resolution of association mapping. The genome-wide LD decay (*r*^2^ < 0.2) in maize is ~ 5.5 kb in maize Hapmap 2 [3] and was estimated to be 1.6 kb in the association panel in the present study, based on the integration of 1.28M SNPs (Additional file 1: Figure S3b). Owing to the rapid LD decay, which confers high mapping resolution, over 60% of the eQTLs attained a resolution level of one candidate gene. About 20% of the eQTLs were resolved to two candidate genes. In a comparison of the eQTLs detected in the present study with those identified in maize kernels (15 days after pollination [16]), only about 12% of the eQTLs overlapped, indicating that gene expression architecture is tissue-dependent and dynamic (Additional file 1: Figure S3c). Approximately 27% of the eQTLs, within the identified 73,573 eQTLs, were static and consistently identified across the three conditions. This provides strong evidence on how these genes are genetically regulated. Notably, 53,040 dynamic eQTLs were identified, capturing an unprecedented number of regulatory variants for drought response in maize seedlings and providing an important foundation for future analyses. Although the decision was made that at least three significant SNPs needed to be detected to identify and designate an eQTL, this does not fully rule out the potential effect of random errors in the quantification of gene expression and result in the overestimation of the number of dynamic eQTLs. Therefore, we recommend that dynamic eQTLs detected under both WS1 and WS2, but not under WW, should be prioritized for subsequent studies pertaining to the regulation of drought-responsive gene expression. Lead SNPs of dynamic local eQTLs were more frequently located in regulatory regions of the candidate gene compared with the lead SNPs in static eQTLs, indicating that stress-responsive sequences are potentially detectable in the up- or downstream of genes associated with dynamic local eQTLs (Additional file 1: Figure S3d).

Distant eQTLs usually act through diffusible factors to fulfill long-range control of gene transcription. In particular, transcription factors serve classic roles as regulatory agents that function as molecular switches of the gene expression at the terminal end of the stress signal transduction pathways associated with stress response [45]. A total of 3733 distant eQTLs were resolved to transcription factors, and a multi-level and multi-family model of a transcriptional regulation hierarchy was constructed based on their regulatory relationships (Fig. 4, Additional file 2: Table S4). To our knowledge, this represents the first comprehensive regulatory network constructed through eQTL analysis, owing to the high resolution of candidate gene detection by GWAS in maize. Previously, the target genes of a given TF were usually identified through analysis of specific gene overexpression or mutant lines and then possibly confirmed by ChIP-qPCR/seq analysis. Although the strategy and results are highly informative, the throughput using this approach is rather low, and more importantly, the interactive or orchestral effects of different types of TFs in an organism cannot be simultaneously considered and evaluated.

An eQTL hotspot was detected on chromosome 6, which is associated with the expression variation of a cluster of 21–24 nearby genes in response to the three water regimes. Notably, this hotspot and the regulated genes were located within a 2.9-Mb presence-absence variation (B73_V4_Chr6: 22.4–25.3 Mb) between the B73 and Mo17 genome [46]. Another eQTL hotspot (B73_V4_Chr2: 120.0–153.1 Mb) was rich in structural variations [47], and the LD in this region was significantly greater than other regions (Additional file 1: Figure S4), indicating that structural variation is an important resource for regional-level LD and expression variation. The growing availability of whole-genome sequences of additional maize accessions will undoubtedly facilitate a better understanding of the nature of the causal polymorphisms underlining eQTLs and eQTL hotspots.

eQTL mapping combined with MR analysis facilitates bridging the gap between genetic and phenotypic associations. In the present study, MR analysis prioritized 97 candidate genes in the regulation of drought response; including 51 positive and 46 negative contributors (regarding gene expression) to maize drought tolerance (Fig. 5, Additional file 2: Table S5). Among them, *ahb2* was verified negatively associated with drought tolerance in maize seedlings. Re-sequencing of the 3.5-kb *abh2* genomic fragment among 141 accessions identified InDel-212 (540 bp in length) which was significantly associated with the gene expression and strong LD with the eQTL lead SNP (SNP2344) (Fig. 6a). InDel-212 was identified as a DTA (*h*AT) family DNA transposon element flanked by an 8-bp target site duplication [48]. It is located 212 bp upstream of the start codon (ATG) and contains one MYB (TGGTTAG) and one MYC binding element (CAATTG), according to the plant *cis*-acting regulatory DNA elements (https://www.dna.affrc.go.jp/PLACE/). Consequently, the presence of these regulatory elements provides evidence to suggest that InDel-212 may play an important role in the regulation of gene expression (Additional file 1: Figure S5a). Further exploration of the 9-kb *abh2* locus between B73 and CIMBL55, a drought-tolerant accession [6, 10], unraveled two additional structure variations. However, when they were genotyped among the 141 accessions, none of them was more significantly associated with the gene expression than InDel-212 under drought stress (Additional file 1: Figure S5b). In addition, several RNAPII- and H3K4me3 chromatin interaction loops were detected in close proximity to InDel-212, based on the chromatin interaction analysis by paired-end tag (ChIA-PET) sequencing in B73 [49]. Taken together, these data collectively suggest that InDel-212, a 540-bp insertion present in drought-susceptible accessions, may be the causative variation of *abh2*.

ABA is involved in a variety of physiological processes during the plant life cycle, including seed dormancy, germination, stomatal movement, fruit development, and responses to biotic and abiotic stresses [45, 50]. A phylogenetic analysis revealed that in several plant species (*Arabidopsis*, rice, and sorghum), ABA 8′-hydroxylase is encoded by multiple genes (Additional file 1: Figure S6a). There are five paralogous genes in the maize genome, of which the two closest homologs, *abh1* and *abh2*, exhibit a similar pattern of tissue expression (Additional file 1: Figure S6b). None of the three knockout alleles of *abh2* displayed any obvious phenotypic abnormalities in regard to the germination or plant development, probably due to the functional redundancy or compensation provided by the paralogous genes. Although modulating endogenous ABA level or response has been supposed to be a promising strategy for plant stress tolerance improvement, it is still challenging to determine, among a number of ABA-related genes, which gene and genetic modification strategy are appropriate to enhance the trait. The present work represents a promising target and strategy. Disrupting *abh2* function or reducing its expression can fine-tune ABA levels in plants subjected to drought for the genetic improvement of plant stress tolerance.

## Conclusions

The regulatory variants controlling the gene expression, constitutively or drought dynamically, were unraveled on a genome-wide scale in maize. It provides significant information that can be used to better understand the role of distal and proximal genetic effects on gene expression regulation. The prioritized candidate genes in drought tolerance are valuable targets for further gene functional study or allelic mining. Gene knockout strategy may be directly applied to genes whose expression is negatively associated with stress tolerance to improve stress tolerance.

## Methods

### Plant accessions and RNA-seq analysis

Two hundred twenty-four maize accessions (inbred lines) were derived from a maize association mapping panel [6, 17], including three additional lines (A188, W22, and X178). Plants were germinated and cultivated as previously described [6]. Three duplicated cultivations were performed for each accession. When the seedlings were 2 weeks old, drought treatment was applied to two duplicated cultivations by withholding water, while keeping one duplication for normal growth. On the ninth and thirteenth days of drought treatment, samples of WS1 and WS2 were harvested, respectively. Samples of WW were harvested on the same day with WS1. WS2 were sampled on a similar time point of a day to avoid possible circadian difference in gene expressions 4 days later. Five-cm leaf sections in the middle of the second leaves were harvested from three plants of each germplasm, pooled, and frozen in liquid nitrogen. The samples were stored at − 80 °C prior to RNA extraction. Total RNA was isolated using TRIzol reagent (Biotopped). Libraries from the resulting total RNA were prepared using the TruSeq paired-end mRNA-Seq kit and protocol from Illumina and sequenced on the Illumina HiSequation 2500 system with Illumina TruSeq SBS v3 reagents as previously described [51]. Fifty-eight biological replicates were randomly placed in the different treatments to estimate the repeatability of the experimental procedures. The average expression correlation coefficient of the biological replicates was 0.83, indicating a reasonable reproducibility of the extensive sampling and expression quantification process.

### Sequencing reads quality control and mapping

After removing reads with low sequencing quality and reads with sequencing adapter, we filtered those samples with sequenced reads number less than 10 million to avoid inaccurate quantitative analysis of unsaturated sequencing. Six hundred twenty-seven high-quality RNA-seq samples of 224 inbred lines under three drought treatments were obtained for the following analysis (WW, *n* = 209; WS1, *n* = 208; WS2, *n* = 210), with additional 58 random biological repeats. All sequenced high-quality reads were mapped to the B73_RefGen_v4 (ftp://ftp.ensemblgenomes.org/pub/plants/release-38/fasta/zea_mays/dna/) maize genome using HISAT2 ver.2.0.5 (with parameters: --dta; --score-min L,-0.6,-0.6) [52, 53]. Finally, only alignments with mapping quality (MQ) > 25 for single-end reads and concordant unique alignments for paired-end reads were kept for further analysis.

### Gene expression quantification and clustering analysis

All mapped reads with MQ > 25 were sorted according to their alignment position on the genome. Each gene was calculated by featureCounts [54], and the differentially expressed genes among different germplasms were identified by the edgeR package [55]. Furthermore, the genes with significant differences in at least 20% of germplasm between any two treatments (false discovery rate (FDR) < 0.05) were regarded as differentially expressed genes (DEGs). The StringTie ver.1.3.4d (with parameters: -G -e -B -o -A) [52, 56] software was used to quantify the gene expression levels by calculating the fragments per kilobase of exon per million fragments mapped (FPKM) to normalize the sequencing depth and gene length. To eliminate the batch effects and systematic variation caused by experimental technique, we normalized the FPKM of all expressed genes of each sample by normalizeQuantiles function of LIMMA package in R. Principal component analysis were performed with the prcomp function in R with setting scale = TRUE. According to the elbow method algorithm, the expression matrix was drawn through the pheatmap package with kmeans_k = 3. We normalized the FPKM values of each gene using a normal quantile transformation (qqnorm function in R). Those genes with normalized FPKM above 0.05 across at least 20% of the accessions were computed for eQTLs detection by a mixed linear model.

### SNP detection and filtering

To identify the genotype of each inbred line, we merged the alignment results (MQ > 25) of three different treatments for each germplasm into one file in bam format and then detected SNPs using the Genome Analysis Toolkit (GATK, version 4.0.5.0) [57]. Firstly, we marked those duplicated reads with the Picard package v1.115 MarkDuplicates (http://broadinstitute.github.io/picard/) and filtered out the spliced mapped reads using SplitNCigarReads package of GATK. Secondly, we performed SNP variant calling by both HaplotypeCaller package of GATK and mpileup package of SAMtools [57, 58], and then we defined the credible variants as those intersection variants that satisfied the following parameters: quality depth (QD) ≥ 2.0 and ReadPosRankSum ≥ − 8.0 and FS ≤ 60.0 and Qual ≥ meanQual. Thirdly, we recalibrated the base quality score based on our defined credible variants using the BaseRecalibrator package of GATK. Lastly, we performed the final SNP variant calling by the HaplotypeCaller and GenotypeGVCFs package of GATK. All detected bi-allelic SNP variants were used for further analysis.

To obtain a high-quality genotype, we firstly filtered out those low-quality SNPs by the GATK VariantFiltration package with the following parameters: --cluster-window-size 35, --cluster-size 3, FS ≤ 30, and QD ≥ 2.0. For each germplasm, if the MAF < 0.05, we considered that it was due to sequencing errors and processed the heterozygous genotypes by modifying the genotype of the minor allele, into its corresponding major allele. Whereas we masked this heterozygous position as missing data. Finally, 1,899,223 SNPs were obtained from our sequenced dataset. To estimate the accuracy of the genotype called from this study, we download another high-quality SNPs dataset derived from 368 inbred lines which is consisting of 1.25M SNPs with a minor allele frequency greater than 0.05 (http://www.maizego.org/Resources.html) [16] and calculated the concordant rate of the genotypes by comparing the overlapping SNP set from the same inbred line. We further integrated our genotype dataset with the 1.25M dataset to increase the SNP maker density, and 2,912,412 SNPs were obtained. After filtering SNPs with missing rate < 0.6 and MAF ≥ 0.05, finally, 1,288,889 SNPs were retained and used for further association analysis.

### GWAS for eQTL identification

eQTL analysis for each expressed gene, whose expression level (FPKM) was above 0.05 in more than 20% accessions (29,614 genes in WW, 30,019 genes in WS1, 30,272 genes in WS2), was computed for GWAS analysis using a linear mixed model [59] by incorporating the kinship coefficients, population structure, and hidden confounding factors, performed by TASSEL (version 5.0) with -mlm -mlmVarCompEst P3D -mlmCompressionLevel None.

The kinship coefficients were estimated by selecting 81,612 uniformly distributed SNPs which was of the lowest missing rate in 10-kb windows, using PHYLIP (version 3.696). The population structure was inferred with fastSTRUCTURE [60]. We further estimated the hidden confounding factors contributing to the expression variability by Bayesian factor analysis (implemented in PEER), and fourteen confounding hidden factors accounting for gene expression variability of each drought treatment were retained and included in the mixed model to examine the validity of association significance, respectively [61, 62]. Benjamini-Hochberg (BH) rejection thresholds were set for WW, *P* < 7.64E−7; WS1, *P* < 7.20E−7; and WS2, *P* < 8.36E−7 (FDR < 0.05). The eQTLs for a specific etrait were identified through three consecutive steps: (1) we grouped all significant SNPs into one eQTL, if their physical distance is < 5 kb; (2) if the SNPs from different eQTLs were within a LD block (*r*^2^ > 0.1), calculated by Haploview with the parameters: -n -maxdistance 250 -minMAF 0.05 -hwcutoff 0 –dprime [63], the eQTLs were combined and the most significant SNP was retained; (3) if multiple eQTLs were detected within one candidate regulatory gene, they were combined and the most significant SNP was retained. Finally, the most significant SNP (lead SNP) was representative of the detected eQTL.

### Regulatory hierarchy network inference

The entire set of available characterized maize transcription factors (www.grassius.org/grasstfdb.php and http://plntfdb.bio.uni-potsdam.de/v3.0/) was used to identify certain TF families. The hierarchy height of TFs was calculated as previously described: *h* = (*O* − *I*)/(*O* + *I*), where *O* and *I* are out-degree and in-degree of examined TF through regulatory relationships, respectively, as described [35].

### Mendelian randomization analysis

MR analysis was performed to reveal the relationship between gene expression level and the phenotype as previously described [21]. In detail, *z* was denoted as the most significantly associated SNP detected by the eQTL analysis under WW, WS1, and WS2 conditions; *x* as the normalized gene expression levels; and *y* as the survival rate of maize seedlings under drought [6]. Then, a two-step least-squares (2SLS) estimate of the effect of *x* on *y* from an MR analysis was *b*_*xy*_ = *b*_*zy*_/*b*_*zx*_, where *b*_*zy*_ and *b*_*zx*_ were the least-squares estimates of *y* and *x* on *z*, respectively, and *b*_*xy*_ was interpreted as the effect size of *x* on *y* free of confounding from non-genetic factors. The sampling variance of the 2SLS estimate was var. (*b*_*xy*_) = $\left[\operatorname{var}(y)\left(1-{R}_{xy}^2\right)\right]/\left[n\operatorname{var}(x){R}_{zx}^2\right]$, where *n* was the sample size, ${R}_{xy}^2$ was the proportion of variance in *y* explained by *x*, and ${R}_{zx}^2$ was the proportion of variance in *x* explained by *z*. We therefore could have a statistic *T*_MR_ = ${b}_{xy}^2/$var. (*b*_*xy*_) to test the significance of *b*_*xy*_, where *T*_MR_ = ${\chi}_1^2$. In total, 30,006 tests (genes with identified eQTLs on the same chromosome) were done, therefore, we set a uniformed experiment-wise significance Bonferroni threshold as *P* < 3.33 × 10^−5^.

### Generation and analysis of *abh2*-CRISPR lines

The CRISPR/Cas9 knockout vector was constructed according to the previously described [64]. The Oligo-F and Oligo-R were annealed and inserted between two BsaI sites of pBUE411. The resulting vector was transformed into *Agrobacterium* strain EHA105, then the *Agrobacterium*-mediated method was used to transform maize immature embryos. Three T_1_ plants with a homozygous mutation at the target sites were self-pollinated to get T_2_ progenies. T_2_ plants were further genotyped by PCR and sequencing to confirm the presence of mutations at the target sites (Additional file 2: Table S6). T_2_ and wild-type plants were used for further analyses. Drought test was performed as described by Wang et al. [6]. At least 18 plants of each line were compared in each test, and statistical analyses were based on the data obtained from four independent experiments. Leaf water loss was determined by a gravimetric method. The second leaves were harvested from three plants of each genotype at the V2 stage. Leaf water loss was determined by allowing detached leaves to desiccate at room temperature (25 °C) and humidity (48%) and weighing every 10 min for the first 2 h, and once an hour to 24 h. Leaf water loss rate was calculated based on gravimetric water loss in relation to fresh weight from three biological replicates.

### Quantification of endogenous ABA content

The planting and drought treatment methods are the same as those of RNA sequencing samples. Five-cm-long leaf sections in the middle of the second leaves were harvested from three plants of each genotype, pooled, and flash-frozen in liquid nitrogen. Fifty milligrams of the fine powder was mixed with 500 μl extracting solution (IPA:H_2_O:HCl = 1000:500:1) and 50 μl internal standard solution (containing 10 ng C_15_H_14_D_6_O_4_) and in a shaking incubation at 4 °C for 0.5 h in 1.5 ml centrifuge tubes. Then, 1 ml of trichloromethane (CHCl_3_) was added and mixed at 4 °C for 0.5 h. The bottom solution (~ 1.2 ml) obtained by centrifugation was blow-dried with nitrogen gas. Dissolved with 100 μl methanol, the supernatant after centrifugation was determined by UPLC-Q Extractive MS in biomass spectrometry laboratory, China Agricultural University (Beijing, China).

## Supplementary information

**Additional file 1: Figure S1.** Population characterization of 224 maize accessions and the number of RNA-seq reads. **Figure S2.** Expression pattern clustering of 15,369 differentially expressed genes (DEGs) in response to drought stress and gene ontology enrichment of each cluster. **Figure S3.** Statistical analysis of dynamic-eQTLs encoding *TF* genes, estimation of the genome-wide linkage disequilibrium (LD) decay, comparison of eQTLs detected in maize kernels and leaves, and the distribution of the lead SNPs of the static and dynamic local-eQTLs. **Figure S4.** Comparison of hotspots, structural variation distribution, and LD on maize chromosomes. **Figure S5.** Analysis of the causative variation of *abh2*. **Figure S6.** Phylogenetic and expression level comparison of *abh2* homologous genes.

**Additional file 2: Table S1.** The SNP concordant rate between the previous and present studies. **Table S2.** Information on static and dynamic eQTLs identified in the present study. **Table S3.** 3733 eQTLs directly located in TF genes. **Table S4.** Detail information on the eQTL network. **Table S5.** Detail information on 97 candidate genes identified by Mendelian Randomization analysis. **Table S6.** Primers used in the present study.

**Additional file 3.** Review history.

## Data Availability

The RNA-seq reads, gene expression level (FPKM) in each sample, and sequence variation data generated in this study have been deposited to the Genome Sequence Archive [65] and Genome Variation Map [66] in BIG Data Center [67], Beijing Institute of Genomics (BIG), Chinese Academy of Science, under accession number (CRA000334 [68], CRA002002 [69] and GVM000048 [70]). The script for the MR analysis can be accessed at GitHub [71]. The RNA-seq reads also have been deposited in the National Center for Biotechnology Information under project number PRJNA637522 [72].
