# Supplementary material for: Mapping regulatory variants controlling gene expression in drought response and tolerance in maize
Source: Genome Biol. 2020 Jul 6;21:163. doi: 10.1186/s13059-020-02069-1 (PMC7336464; doi:10.1186/s13059-020-02069-1)
Supplement: Supplementary file 1 — Additional file 1: Figure S1. Population characterization of 224 maize accessions and the number of RNA-seq reads. Figure S2. Expression pattern clustering of 15,369 differentially expressed genes (DEGs) in response to drought stress and gene ontology enrichment of each cluster. Figure S3. Statistical analysis of dynamic-eQTLs encoding TF genes, estimation of the genome-wide linkage disequilibrium (LD) decay, comparison of eQTLs detected in maize kernels and leaves, and the distribution of the lead SNPs of the static and dynamic local-eQTLs. Figure S4. Comparison of hotspots, structural variation distribution, and LD on maize chromosomes. Figure S5. Analysis of the causative variation of abh2. Figure S6. Phylogenetic and expression level comparison of abh2 homologous genes. [file 13059_2020_2069_MOESM1_ESM.pdf]

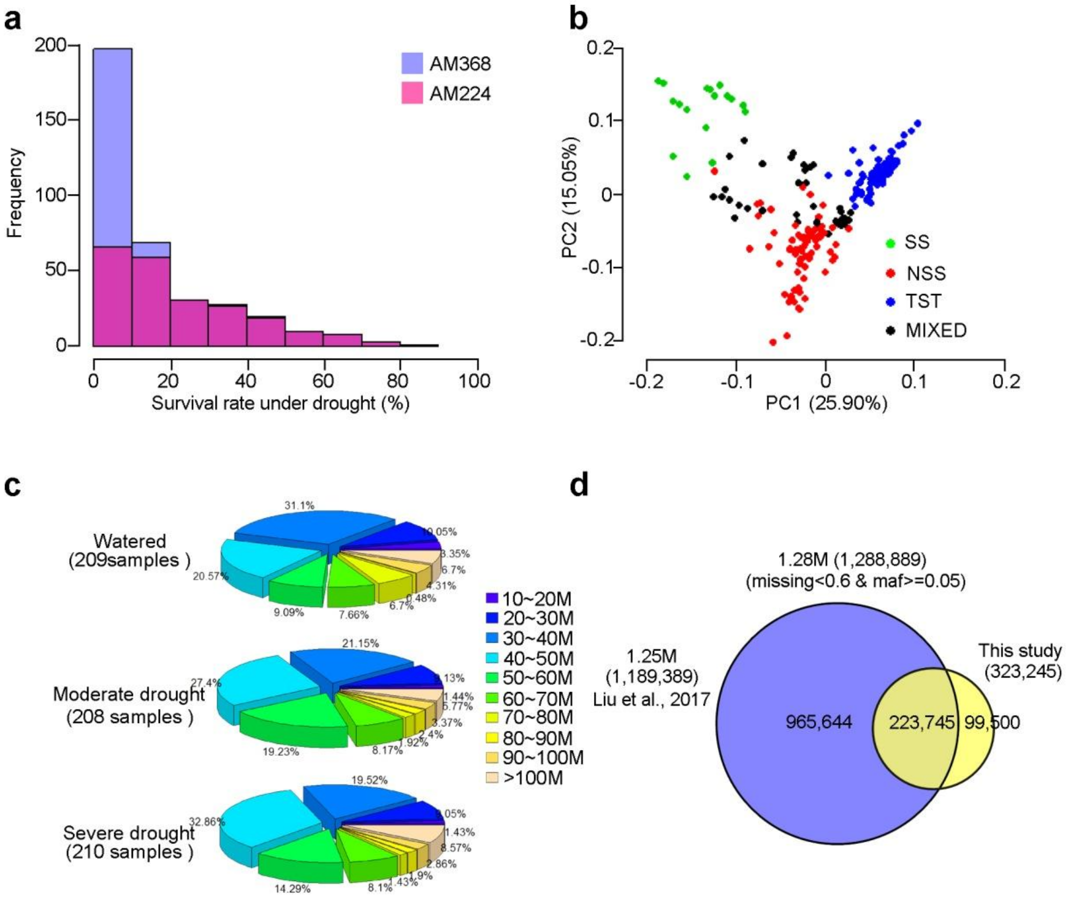

Figure S1. Population characterization of 224 maize accessions and the number of RNA-seq reads. (a) Distribution of drought tolerance referring to rate of plant survival rate under severe drought stress, based on the phenotypic data published by Wang et al., 2016 in the original 368 association panel and this experimental population [6]. (b) PCA analysis of 224 maize inbred lines with 1.28M SNPs. (c) Distribution of the number of unique mapping reads of the 627 RNA-seq samples in the experimental population. (d) Venn diagram illustrating the new 1.28M SNP dataset obtained through integrating the previously reported 1.25M SNPs [16] and the 323,245 SNPs identified in the present study.

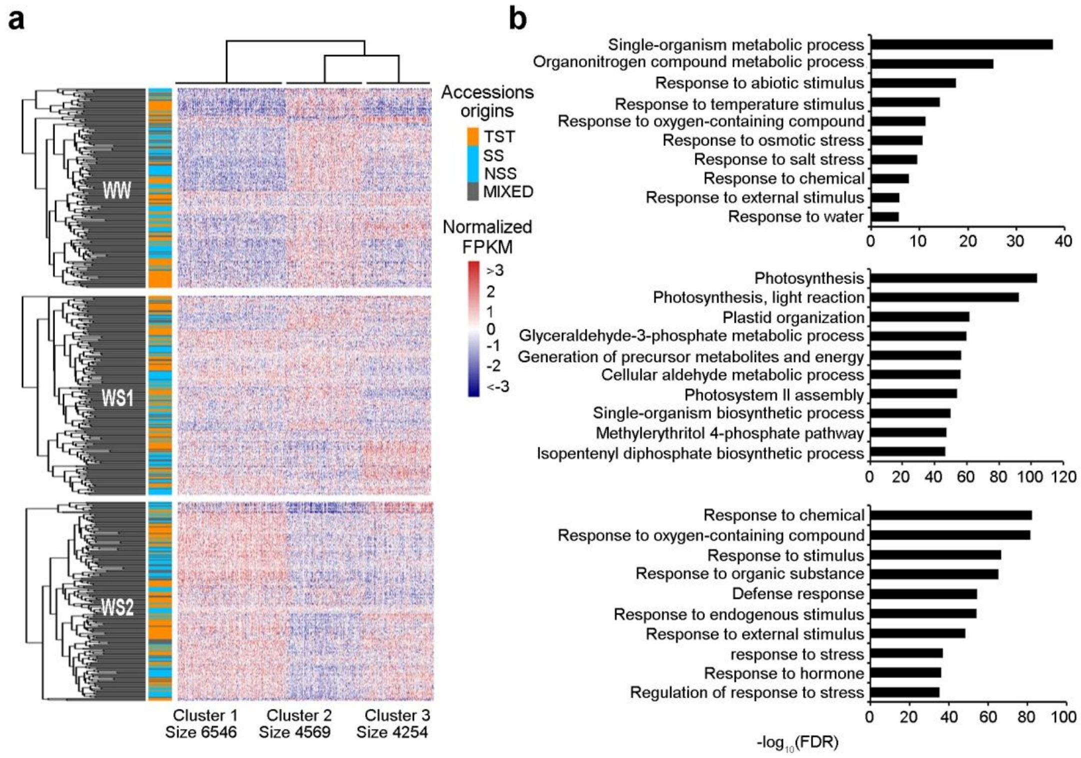

Figure S2. Expression pattern clustering of 15,369 differentially expressed genes (DEGs) in response to drought stress and gene ontology enrichment of each cluster. (a) Three clusters of 15,369 DEGs responsive to drought stress. The FPKM values of each gene over all the samples were normalized through a qqnorm function in R. Normalized expression levels of 15,369 genes sorted into 3 clusters by k-means clustering algorithm. Cluster 1: drought-inducible genes; Cluster 2: drought-repressed genes; Cluster 3: response-variable genes. The hierarchies of all the accessions were constructed based on the gene expression levels of 15,369 DEGs under three conditions. Color of the bar indicates the level of normalized gene expression. The origin of maize accessions is indicated by orange for TST (Tropical/Subtropical), blue for stiff stock and non-stiff stock (SS and NSS), and grey for mixed origins. (b) Gene ontology enrichment of biological pathways for the three clusters using AgriGO (http://bioinfo.cau.edu.cn/agriGO/) based on 3 clusters. The top 10 highest enriched for each cluster are displayed.

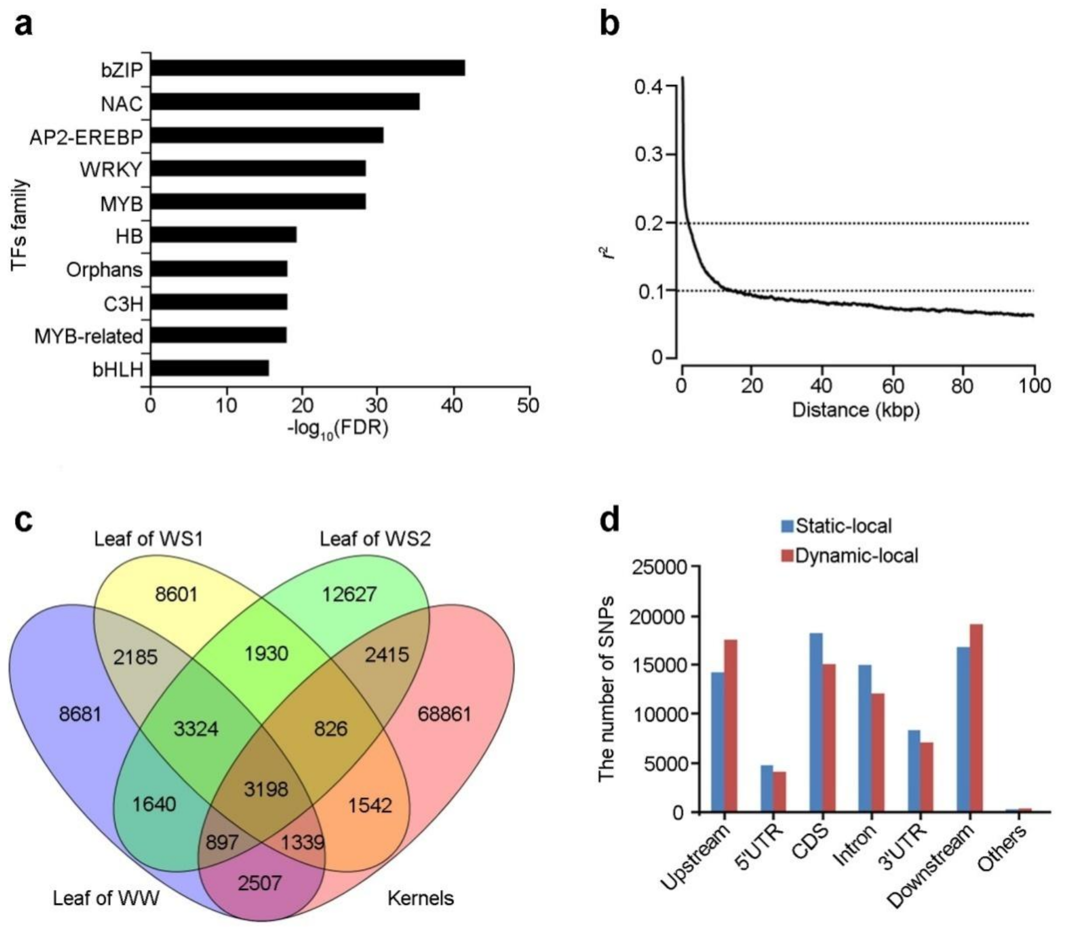

Figure S3. Statistical analysis of dynamic-eQTLs encoding *TF* genes, estimation of the genome-wide linkage disequilibrium (LD) decay, comparison of eQTLs detected in maize kernels and leaves, and the distribution of the lead SNPs of the static and dynamic local-eQTLs. (a) Enrichment of *TF* gene families based on dynamic-eQTLs encoding *TF* genes. The significance was calculated using phyper function in R and adjusted by Bonferroni correction, with all *TF* genes annotated in B73_V4 as background. (b) The genome-wide LD decay was calculated based on the newly obtained 1.28M SNPs among the 224 maize accessions. LD decay was determined by correlation coefficient (r^2) of SNPs against their distance in the maize genome. Average r^2 was calculated with a 1000-kb window-size with step length of 100-bp by plink1.09. LD generally decayed (r^2 to 0.2) within 1.6-kb in this experimental population. (c) Venn diagrams of eQTLs identified in maize kernels at 15-days after pollination [16] and in leaves under normal and two water stress conditions. (d) Location of the lead SNPs in the candidate genes of the static and dynamic local-eQTLs.

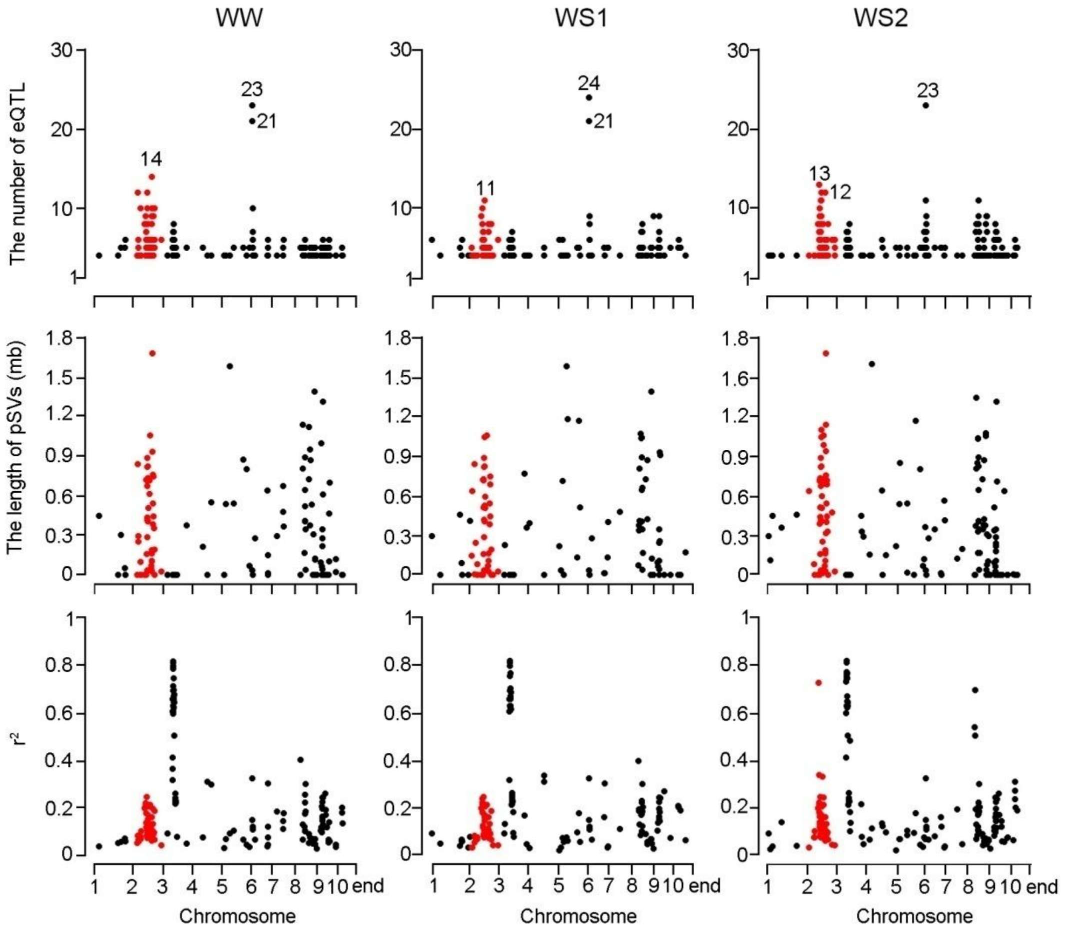

Figure S4. Comparison of hotspots, structural variation distribution, and LD on maize chromosomes. From top to bottom, the y-axis represents the number of eQTLs, cumulative length of structural variation [47] and the regional LD decay determined by correlation coefficient (r^2). The eQTLs in this region (B73_V4_Chr2:120.0-153.1 Mb) are highlighted in red.

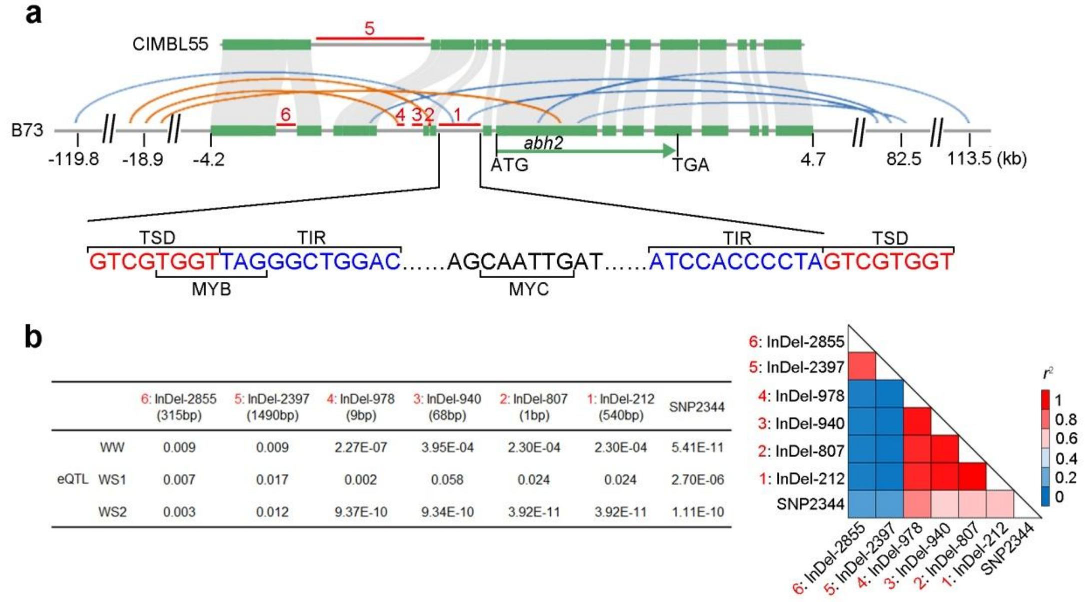

		6: InDel-2855 (315bp)	5: InDel-2397 (1490bp)	4: InDel-978 (9bp)	3: InDel-940 (68bp)	2: InDel-807 (1bp)	1: InDel-212 (540bp)	SNP2344
	WW	0.009	0.009	2.27E-07	3.95E-04	2.30E-04	2.30E-04	5.41E-11
eQTL	WS1	0.007	0.017	0.002	0.058	0.024	0.024	2.70E-06
	WS2	0.003	0.012	9.37E-10	9.34E-10	3.92E-11	3.92E-11	1.11E-10

Figure S5. Analysis of the causative variation of *abh2*.

(a) Synteny of the genomic region of *abh2* between B73 and CIMBL55. Homologous regions are indicated by green rectangle and the sequence synteny is shaded in grey. The location of the start codon (ATG) is labeled as '+1'. Red numbers indicated the six InDels upstream of *abh2*. InDels: 1-4 are also shown in Figure 6a. InDels: 5 and 6 are identified by the genomic sequence synteny analysis, and their genotypes in the 141 accessions were determined by PCR analyses. Blue and orange link indicating chromatin interaction loop detected by anti-H3K4me3 and anti-RNAPII, respectively, based on Peng Y, et al., 2019 [49]. The TSD (target site direct repeat) and TIR (terminal inverted repeat) of InDel-212 (a DTA transposon element) inserted in B73 was identified based on Su et al., 2019 [48]. The MYB and MYC binding sequence are detected according to Plant Cis-acting Regulatory DNA Elements (PLACE) database. (b) The association significance of the six InDels with the *abh2* expression under WW, WS1 and WS2, and the pairwise LD analysis of the six InDels and the original identified eQTL lead SNP (SNP2344). InDel: 1 and 2 are complete LD. The length of each InDel is indicated in parentheses.

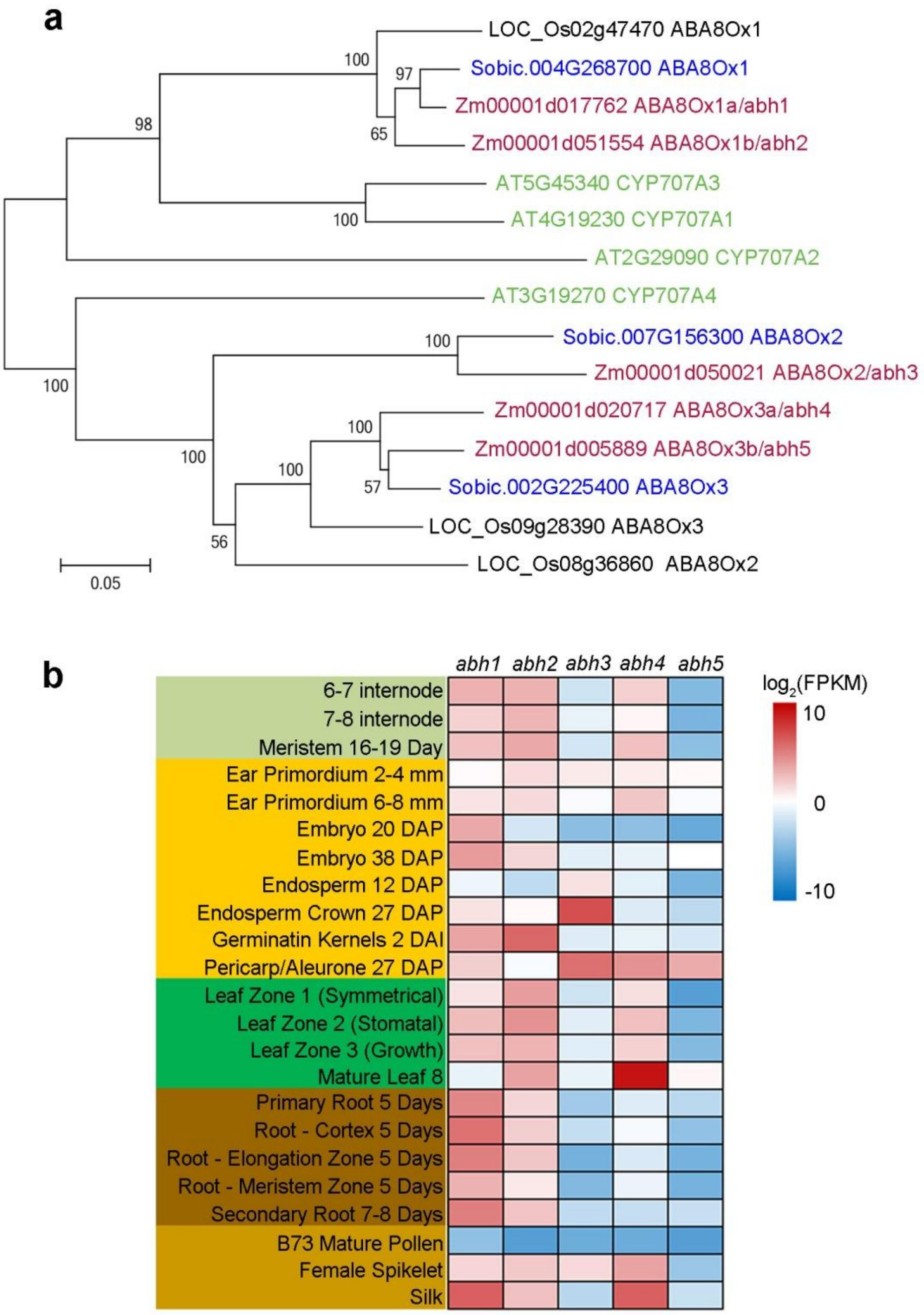

Figure S6. Phylogenetic and expression level comparison of *abh2* homologous genes. (a) Phylogenetic tree of *abh2* genes in maize (red), rice (black), sorghum (blue), and Arabidopsis (green). (b) Expression pattern of *abh* genes in twenty-three different tissues at various plant developmental stages of B73 from MaizeGDB (https://www.maizegdb.org/).
