# Supplementary material for: Mapping regulatory variants controlling gene expression in drought response and tolerance in maize
Source: Genome Biol. 2020 Jul 6;21:163. doi: 10.1186/s13059-020-02069-1 (PMC7336464; doi:10.1186/s13059-020-02069-1)
Supplement: Supplementary file 3 — Additional file 3. Review history. [file 13059_2020_2069_MOESM3_ESM.docx]

Review History

**First round of review**

**Reviewer 1**

**Are you able to assess all statistics in the manuscript, including the appropriateness of statistical tests used?**

Yes, and I have assessed the statistics in my report.

**Comments to author:**

The manuscript described the generation of RNA-Seq dataset in 224 maize accessions and drought-related eQTL mapping on maize genes, which provided an important resource for the community. One candidate gene was further validated through CRISPR-CAS9. Generally, this is a very nice work. I only have three minor suggestions for the paper:

1. What about the causative variation for abh2? Any associated variant located in key motifs of abh2 promoter region? The authors sequenced 3.5-kb genomic region of abh2 among 141 maize inbred lines and found all the significant variants were synonymous or locating in non-coding regions. Further analyses and experiments on the regulation region of abh2 are needed.
2. The authors used RNA-Seq reads for genotype confirmation and also to complement previously published. In Method section, "To estimate the accuracy of the genotype called from this study, we download another high quality SNPs dataset derived from 368 inbred lines which is consisting of 1.25M SNPs".  However, I do not find the accuracy (or called as the concordant rate) throughout the manuscript. As an important index for the genotype data quality (particularly, any lines are inconsistant with previous ones due to heterozygous issues or error in planting and distributing), a list of the accuracy for all the 224 maize accessions should be estimated and provided in the paper.
3. The RAW data of RNA-Seq and the resulting FPKM table should be available in public database and/or supplementary files.

**Reviewer 2**

**Are you able to assess all statistics in the manuscript, including the appropriateness of statistical tests used?**

Yes, and I have assessed the statistics in my report.

**Comments to author:**

This work generated 627 RNA-seq from 224 core maize accessions which represent a wide genetic diversity in three water regimes (WW, WS1, WS2). The expression level of each gene was treated as a quantitative trait, and GWAS analysis was performed based on 1.28 M SNPs to identify eQTLs. On the other hand, by integrating previous phenotypic measurements of drought tolerance of 224 maize accessions and newly obtained expression profiles in this study, the authors performed Mendelian Randomization test (MR) to identify 97 candidate genes for future drought resistance improvement in maize.
eQTL identification by GWAS has been used to investigate kernel size-related genes during 5 and 15 days after pollination (DAP) (Pang et al., 2019), and this method has not been used in stress response. This work creatively classified eQTLs into static and dynamic catogories, the former refers to eQTLs that were identified in all three water regimes, while the latter refers to those identified only in one or two water regimes. Since the LD decay is low with 1.28 M SNPs, over 60% eQTLs attained a resolution level of one-candidate-gene, allowing for construction of transcription network using candidate TFs and 44 selected drought response genes. Finally, 97 candidate genes for drought response were identified using MR test. One of these genes, abh2, was shown to be negatively associated with drought tolerance by gene-knock-out strategy. Overall, it is a very nice work, and the manuscript can be of broader interest, especially for the scientists working in plant biology and agriculture. However, I have a few concerns with the data and methodology in the manuscript that the authors need to address.

1) ABA-related genes were divided into three clusters by k-means clustering algorithm, genes in cluster 1 and 2 are consistently increase or decrease with drought in most varieties, while cluster 3 exhibits great variation between varieties (Figure 1). The authors hold the opinion that genes in cluster 3 may underlie different drought tolerance of maize accessions (page 7), however, there is possibility that genes in cluster 1 or 2 are also involved in drought tolerance, with diverse extent of increase or decrease.

2) As mentioned in discussion (page 14), dynamic eQTLs detected under both WS1 and WS2, but not under WW may be more correlated with drought tolerance. But the definition of dynamic eQTLs in results is somehow vague (page 7).

3) Transcription network has been constructed with dozens of candidate TFs in eQTLs and their potential target genes (44 etraits), it is said that ten of the distant regulatory activities (MYB, NAC, bZIP, C2H2 and HB TF family genes) were identified in Arabidopsis. However, there is a lack of ChIP-qPCR validation of these interactions in maize.

4) It is said that over 60% of eQTLs attained a resolution level of one-candidate-gene, so less than 40% of eQTLs are located in non-coding regions, such as promoters, intergenic regions, intron regions. This implies that transcription level of genes is mostly regulated by other upstream genes, rather than by non-coding elements throughout genome, is it correct?

5) Is there any eQTLs that control the expression of abh2?

6) Which SNPs or indels that underlie the transcription level variations of abh2 in 224 maize accessions?

7) There are totally 97 candidate genes, have you verified the drought tolerance change of other genes except for abh2?


Pang, J., Fu, J., Zong, N., Wang, J., Song, D., Zhang, X., He, C., Fang, T., Zhang, H., Fan, Y., et al. (2019). Kernel size-related genes revealed by an integrated eQTL analysis during early maize kernel development. Plant Journal 98, 19-32.

**Authors Response**

**Point-by-point responses to the reviewers’ comments:**

Comment

*Response*

Reviewer #1: The manuscript described the generation of RNA-Seq dataset in 224 maize accessions and drought-related eQTL mapping on maize genes, which provided an important resource for the community. One candidate gene was further validated through CRISPR-CAS9. Generally, this is a very nice work. I only have three minor suggestions for the paper:
*Response: Thanks for the positive comments on the overall results. According the suggestions, we revised the manuscript. All the changes in the revised manuscript were highlighted in RED. We hope the revision is satisfactory. The following is the point-by-point response:*

1. What about the causative variation for abh2? Any associated variant located in key motifs of abh2 promoter region? The authors sequenced 3.5-kb genomic region of abh2 among 141 maize inbred lines and found all the significant variants were synonymous or locating in non-coding regions. Further analyses and experiments on the regulation region of abh2 are needed.
*Response: In order to answer this question, firstly we carefully analyzed all the newly identified variations from the re-sequenced 3.5-kb genomic region of abh2 among 141 maize inbred lines. We found that InDel-212, 540-bp in length, was identified as a DTA (hAT) family DNA transposon element flanked by an 8-bp target site duplication [48] (Additional file1: Figure S5a). Secondly, it locates 212-bp upstream of the start coden (ATG) of abh2 and contains one MYB binding element (TGGTTAG) and one MYC binding element (CAATTG), according to Plant Cis-acing Regulatory DNA Elements (https://www.dna.affrc.go.jp/PLACE/). Consequently, the presence of these regulatory elements provides evidence to suggest that InDel-212 may play an important role in the regulation of gene expression (Additional file1: Figure S5a). Thirdly, we compared the 9-kb genomic sequence at abh2 locus between B73 and CIMBL55, a drought tolerant inbred line [6, 10]. Two additional structure variations unraveled, however, when they were genotyped among the 141 accessions, none of them was more significantly associated with the gene expression than InDel-212 under drought stress (Additional file1: Figure S5b). In addition, several chromatin interaction loops were detected by anti-RNAPII and anti-H3K4me3 in close proximity to InDel-212. Taken together, these data collectively suggest that InDel-212, a 540-bp insertion present in drought susceptible accessions, may be the causative variation of abh2.
To address this question, we a new supplemental figure (Additional file 1: Figure S5), was appended (the original Additional file 1: Figure S5 was changed into Additional file 1: Figure S6), and the description was added in Discussion, from Page 15, Line 13 to Page 16, Line 2. It reads: "Re-sequencing of the 3.5-kb abh2 genomic fragment among 141 accessions identified InDel-212 (540-bp in length) which was significantly associated with the gene expression and strong LD with the eQTL lead SNP (SNP2344) (Figure 6a). InDel-212 was identified as a DTA (hAT) family DNA transposon element flanked by an 8-bp target site duplication [48]. It is located 212-bp upstream of the start codon (ATG) and contains one MYB (TGGTTAG) and one MYC binding element (CAATTG), according to the Plant Cis-acing Regulatory DNA Elements (https://www.dna.affrc.go.jp/PLACE/). Consequently, the presence of these regulatory elements provides evidence to suggest that InDel-212 may play an important role in the regulation of gene expression (Additional file1: Figure S5a). Further exploration of the 9-kb abh2 locus between B73 and CIMBL55, a drought tolerant accession [6, 10], unraveled two additional structure variations. However, when they were genotyped among the 141 accessions, none of them was more significantly associated with the gene expression than InDel-212 under drought stress (Additional file1: Figure S5b). In addition, several RNAPII- and H3K4me3 chromatin interaction loops were detected in close proximity to InDel-212, based on the chromatin interaction analysis by paired-end tag (ChIA-PET) sequencing in B73 [49]. Taken together, these data collectively suggest that InDel-212, a 540-bp insertion present in drought susceptible accessions, may be the causative variation of abh2."*

2. The authors used RNA-Seq reads for genotype confirmation and also to complement previously published. In Method section, "To estimate the accuracy of the genotype called from this study, we download another high quality SNPs dataset derived from 368 inbred lines which is consisting of 1.25M SNPs". However, I do not find the accuracy (or called as the concordant rate) throughout the manuscript. As an important index for the genotype data quality (particularly, any lines are inconsistant with previous ones due to heterozygous issues or error in planting and distributing), a list of the accuracy for all the 224 maize accessions should be estimated and provided in the paper.
*Response: This is a good point. We totally agree with this review's comment that genotyping data quality is of vital importance for the following studies. Actually, we had carefully analyzed the condordant rate between our SNP identification with the previously published 1.25M data, based on the common sites identified in both studies. The average concordant rate was 93.7%. To avoid any uncertainty, we designated the sites as ‘data missing’ where inconsistent genotype data were observed. After filtered with the criteria of missing rate < 0.6 and minor allele frequency ≥ 5%, 1,288,889 SNPs were obtained for these 224 accessions which were used for further analysis. For clarity, a new supplemental table (Additional file 2: Table S1) was added for the detail information for each accession. The numbers of original supplemental tables were changed accordingly. The description was added in Result, Page 5, Line 23-26. It reads: "The average concordant rate of the genotyping data of this study and the previous work was 93.7% (Additional file 2: Table S1). To avoid any uncertainty, we designated the sites as ‘data missing’ where inconsistent genotype data were observed."*

3. The RAW data of RNA-Seq and the resulting FPKM table should be available in public database and/or supplementary files.
*Response: Thanks for this comment. For clarity, we clarified this point in Availability of Data and Materials, Page 28, Line 9-10. It reads: "The RNA-seq reads, gene expression level (FPKM) in each sample and sequence variation data generated in this study have been deposited to the Genome Sequence Archive [65] and Genome Variation Map [66] in BIG Data Center [67], Beijing Institute of Genomics (BIG), Chinese Academy of Science under accession number (CRA000334 [68], CRA002002 [69] and GVM000048 [70]). The script for the MR analysis can be accessed at GitHub [71]."*

Reviewer #2: This work generated 627 RNA-seq from 224 core maize accessions which represent a wide genetic diversity in three water regimes (WW, WS1, WS2). The expression level of each gene was treated as a quantitative trait, and GWAS analysis was performed based on 1.28 M SNPs to identify eQTLs. On the other hand, by integrating previous phenotypic measurements of drought tolerance of 224 maize accessions and newly obtained expression profiles in this study, the authors performed Mendelian Randomization test (MR) to identify 97 candidate genes for future drought resistance improvement in maize.
eQTL identification by GWAS has been used to investigate kernel size-related genes during 5 and 15 days after pollination (DAP) (Pang et al., 2019), and this method has not been used in stress response. This work creatively classified eQTLs into static and dynamic catogories, the former refers to eQTLs that were identified in all three water regimes, while the latter refers to those identified only in one or two water regimes. Since the LD decay is low with 1.28 M SNPs, over 60% eQTLs attained a resolution level of one-candidate-gene, allowing for construction of transcription network using candidate TFs and 44 selected drought response genes. Finally, 97 candidate genes for drought response were identified using MR test. One of these genes, abh2, was shown to be negatively associated with drought tolerance by gene-knock-out strategy. Overall, it is a very nice work, and the manuscript can be of broader interest, especially for the scientists working in plant biology and agriculture. However, I have a few concerns with the data and methodology in the manuscript that the authors need to address.
*Response: Thanks for the positive comments on the work. According the suggestions, we revised the manuscript. All the changes in the revised manuscript were highlighted in RED. We hope the revision is satisfactory. The following is the point-by-point response.*

1) ABA-related genes were divided into three clusters by k-means clustering algorithm, genes in cluster 1 and 2 are consistently increase or decrease with drought in most varieties, while cluster 3 exhibits great variation between varieties (Figure 1). The authors hold the opinion that genes in cluster 3 may underlie different drought tolerance of maize accessions (page 7), however, there is possibility that genes in cluster 1 or 2 are also involved in drought tolerance, with diverse extent of increase or decrease.
*Response: Thanks for this comment. We completely agree with this reviewer that cluster 1 or 2 genes are also possibly involved in drought tolerance with different extent of expression increase or decrease. We did not want to ignore the involvement of the other two cluster genes, but just pointed that the diversity in response direction to drought of cluster 3 genes is important and interesting. To avoid the confusion, we revised the description in Page 7, Line 3-6. It reads: "The diversity in response intensity and direction indicated that natural variants underlie the expression of these genes and potentially contribute to the variation of drought response among these accessions"*

2) As mentioned in discussion (page 14), dynamic eQTLs detected under both WS1 and WS2, but not under WW may be more correlated with drought tolerance. But the definition of dynamic eQTLs in results is somehow vague (page 7).
*Response: Throughout the work, the dynamic eQTLs were designated as those were detected in only under one or two conditions, and those could be detected under three conditions were regarded as static eQTLs. However, in Discussion, we just suggest a study priority on the dynamic eQTLs (detected under both WS1 and WS2, but not under WW) for drought-responsive gene regulation study, among all the identified dynamic eQTLs. For clarity, we revised the description in Discussion in Page 14, Line 5-7. It reads: "Therefore, we recommend that dynamic eQTLs detected under both WS1 and WS2, but not under WW, should be prioritized for subsequent studies pertaining to the regulation of drought-responsive gene expression"*

3) Transcription network has been constructed with dozens of candidate TFs in eQTLs and their potential target genes (44 etraits), it is said that ten of the distant regulatory activities (MYB, NAC, bZIP, C2H2 and HB TF family genes) were identified in Arabidopsis. However, there is a lack of ChIP-qPCR validation of these interactions in maize.
*Response: So far the ChIP-qPCR validation is unavailable in maize, due to the absent of effective antibody of these TFs. Alternatively, the tagged-TF transgenic maize should be generated to carried out the ChIP-qPCR experiment using the commercial tag antibody. We will construct such experiments in later studies for individual candidate gene validation to provide the direct evidence.*

4) It is said that over 60% of eQTLs attained a resolution level of one-candidate-gene, so less than 40% of eQTLs are located in non-coding regions, such as promoters, intergenic regions, intron regions. This implies that transcription level of genes is mostly regulated by other upstream genes, rather than by non-coding elements throughout genome, is it correct?
*Response: We would like to respectfully clarify our viewpoint regarding this interpretation here. The statement: "Over 60% of eQTLs attained a resolution level of one-candidate-gene" was intending to indicate that 60% of the detected eQTL intervals contain a single candidate gene and was referring to the resolution of the eQTL interval. The resolution of an association analysis depends on the genetic linkage disequilibrium (LD). By taking the advantage of the quick LD decay in maize genomes, the resolution of association mapping can usually reach to single candidate gene level. Whereas, the eQTL mapping approach, which uses 200-300 offspring from a cross of two parental lines in which the eQTL interval was normally several Mb, would be hardly able to resolve to the single candidate gene level. Thus, this is an important advantage in comparison to previous eQTL studies based on genetic recombination analysis of a two-parental recombinant population. On the other hand, about 40% of the eQTLs which could not be resolved to a single candidate gene meant that more than one candidate gene resides within the eQTL intervals. As a result, we were not able to decide which one was the most likely candidate gene. However, this was not meant to indicate that these eQTLs were located in non-coding regions. Moreover, the eQTL resolved to a single candidate gene actually meant that all genetic variation within the LD block (where the lead SNP was located) were the possible regulatory elements of the etrait gene, but not only the coding region of the candidate gene. Meanwhile, it is worthwhile to note that among the over 60% of eQTLs (resolved to the level of a single candidate gene), if they were identified as local eQTLs (the lead SNP was 20-kb away from the etrait gene), in most cases they were resolved to the etrait gene itself, since the average intergenic region is estimated to be approximately 45-kb in the maize genome. In these cases, the non-coding sequences were especially of regulatory importance.*

5) Is there any eQTLs that control the expression of abh2?
*Response: Yes. As it was described in Results, Page12, Line 23-25. "Haplotype 1 containing SNP2344 (C-genotype), the lead SNP identified as the abh2 local-eQTL, had significantly higher abh2 gene expression than other haplotypes; supporting that local genetic variants affect abh2 expression (Fig. 5b)."*

6) Which SNPs or indels that underlie the transcription level variations of abh2 in 224 maize accessions?
*Response: This question was also raised by the other reviewer. In order to answer this question, firstly we carefully analyzed all the newly identified variations from the re-sequenced 3.5-kb genomic region of abh2 among 141 maize inbred lines. We found that InDel-212, 540-bp in length, was identified as a DTA (hAT) family DNA transposon element flanked by an 8-bp target site duplication [48] (Additional file1: Figure S5a). Secondly, it locates 212-bp upstream of the start coden (ATG) of abh2 and contains one MYB binding element (TGGTTAG) and one MYC binding element (CAATTG), according to Plant Cis-acing Regulatory DNA Elements (https://www.dna.affrc.go.jp/PLACE/). Consequently, the presence of these regulatory elements provides evidence to suggest that InDel-212 may play an important role in the regulation of gene expression (Additional file1: Figure S5a). Thirdly, we compared the 9-kb genomic sequence at abh2 locus between B73 and CIMBL55, a drought tolerant inbred line [6, 10]. Two additional structure variations unraveled, however, when they were genotyped among the 141 accessions, none of them was more significantly associated with the gene expression than InDel-212 under drought stress (Additional file1: Figure S5b). In addition, several chromatin interaction loops were detected by anti-RNAPII and anti-H3K4me3 in close proximity to InDel-212. Taken together, these data collectively suggest that InDel-212, a 540-bp insertion present in drought susceptible accessions, may be the causative variation of abh2.
To address this question, we a new supplemental figure (Additional file 1: Figure S5), was appended and the description was added in Discussion, from Page 15, Line 13 to Page 16, Line 2. It reads: "Re-sequencing of the 3.5-kb abh2 genomic fragment among 141 accessions identified InDel-212 (540-bp in length) which was significantly associated with the gene expression and strong LD with the eQTL lead SNP (SNP2344) (Figure 6a). InDel-212 was identified as a DTA (hAT) family DNA transposon element flanked by an 8-bp target site duplication [48]. It is located 212-bp upstream of the start codon (ATG) and contains one MYB (TGGTTAG) and one MYC binding element (CAATTG), according to the Plant Cis-acing Regulatory DNA Elements (https://www.dna.affrc.go.jp/PLACE/). Consequently, the presence of these regulatory elements provides evidence to suggest that InDel-212 may play an important role in the regulation of gene expression (Additional file1: Figure S5a). Further exploration of the 9-kb abh2 locus between B73 and CIMBL55, a drought tolerant accession [6, 10], unraveled two additional structure variations. However, when they were genotyped among the 141 accessions, none of them was more significantly associated with the gene expression than InDel-212 under drought stress (Additional file1: Figure S5b). In addition, several RNAPII- and H3K4me3 chromatin interaction loops were detected in close proximity to InDel-212, based on the chromatin interaction analysis by paired-end tag (ChIA-PET) sequencing in B73 [49]. Taken together, these data collectively suggest that InDel-212, a 540-bp insertion present in drought susceptible accessions, may be the causative variation of abh2."*

7) There are totally 97 candidate genes, have you verified the drought tolerance change of other genes except for abh2?
*Response: We are currently of great interest to validate the other candidate genes identified through MR analysis to find out whether their variations play roles in maize drought tolerance. Intensive genetic variation analyses and transgenic work of several candidate genes are being undertaken.

Two references were appended:
[48]. Su W, Gu X, Peterson T. TIR-Learner, a New Ensemble Method for TIR Transposable Element Annotation, Provides Evidence for Abundant New Transposable Elements in the Maize Genome. Mol Plant. 2019; 12:447-60.
[49]. Peng Y, Xiong D, Zhao L, Ouyang W, Wang S, Sun J, Zhang Q, Guan P, Xie L, Li W, et al. Chromatin interaction maps reveal genetic regulation for quantitative traits in maize. Nat Commun. 2019; 10:2632.*

**Second round of review**

**Reviewer 1**

My concerns were all well addressed in the revision. I'm satisfied with the revision.

**Reviewer 2**

The authors addressed all my concerns. No further questions.
